# Hydrogenation Kinetics Study: Precise Control of C=C Bonds in Polyisoprene (PI)-Containing Block Copolymers via Diimide Hydrogenation

**DOI:** 10.1021/acsapm.6c01388

**Published:** 2026-06-03

**Authors:** Luis Felipe Caspari Thiele, Yongha Kim, Corey A. Roberts, Shubhra Goel, Andrew L. Zydney, Ralph H. Colby, Manish S. Kelkar, Uwe Beuscher, Hee Jeung Oh

**Affiliations:** † Department of Materials Science and Engineering, 311285 The Pennsylvania State University, University Park, Pennsylvania 16802, United States; ‡ Department of Chemical Engineering, 311285 The Pennsylvania State University, University Park, Pennsylvania 16802, United States; § Department of Chemistry, The University of Kentucky, Lexington, Kentucky 40506, United States; ∥ AbbVie, North Chicago, Illinois 60064, United States; ⊥ W. L. Gore & Associates, Newark, Delaware 19711, United States; # Institute of Energy and Environment, 311285 The Pennsylvania State University, University Park, Pennsylvania 16802, United States; ∇ Advanced Manufacturing and Design, 311285 The Pennsylvania State University, University Park, Pennsylvania 16802, United States

**Keywords:** polydiene, polyisoprene, hydrogenation, polystyrene−*b*−polyisoprene−*b*−polystyrene triblock copolymer, *p*-toluenesulfonyl hydrazide (TSH), diimide

## Abstract

Polydienes containing a C=C bond in each unit, such as polybutadiene (PBD)- or polyisoprene (PI)-containing polymers, have served as an important backbone of polymer applications in our daily lives. For versatile use of polydienes in various applications, C=C bonds in polydienes can be hydrogenated to achieve a desirable saturation level and/or additionally modified to obtain suitable structure–property sets. Among different hydrogenation procedures, non-catalytic, diimide hydrogenation using *p*-toluenesulfonyl hydrazide (TSH) can enable easier and precise control of hydrogenation level in C=C bonds at mild conditions. Here, for the first time, we reported a systematic hydrogenation kinetics study of one representative PI-containing polymer, i.e., polystyrene-*b*-polyisoprene-*b*-polystyrene (SIS) triblock copolymer with high molecular weight via TSH-based, diimide hydrogenation using NMR, SEC, and thermal analyses. We evaluated hydrogenation level (HL = 0∼99.9%) vs reaction time (0∼24 h) of SIS polymer and compared our kinetics results with other PBD- or PI-containing polymers with different chain architectures, compositions, and hydrogenation conditions to develop a universal efficacy of this diimide hydrogenation method and build a comprehensive understanding among these reports. We achieved HL = 51% in 1 h and HL > 99.9% by 24 h. Our analyses confirm that no side reactions occur up to 48 h without affecting other copolymer blocks. Our kinetics data align well with other similar polydienes but cover a wider range of reaction times (up to 48 h) than those in the literature. Using this information, a target hydrogenation level can be precisely achieved by controlling reaction time under mild conditions. Overall, this TSH-based diimide hydrogenation method is universal and effective over a broad range of PBD- and PI-containing polymers with different chain architectures and compositions.

## Graphical abstract

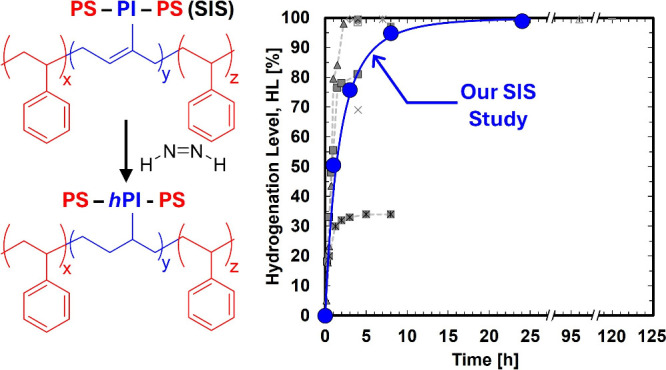

## 1 Introduction

Polydienes, containing a C=C bond in each unit as polymerized from conjugated diene monomers, have served as an important backbone of polymer applications in our daily lives since the 19th century.^1,2^ The most common polydienes include polybutadiene (PBD)-, polyisoprene (PI)-, and poly­(chloroprene)-containing polymers and recently studied bio-based polydienes such as poly­(β-myrcene) and polyphenylbutadiene. In particular, PBD- and PI-containing polymers have been extensively used in both conventional applications such as tire manufacturing, adhesives, packaging, coating, footwear, and industrial components, as well as in emerging applications including separations, power generation, environmental remediation and health-related devices.^3–13^

Versatile benefits of polydienes come from a C=C bond in each unit, which (1) can provide flexibility and elasticity with resilience and (2) can offer reaction sites for hydrogenation (to saturate C=C bonds), cross-linking (to increase mechanical stability), and/or chemical functionalization to achieve a desirable structure–property set for a target application.^1,2,14–19^ For effective and versatile use of polydienes in various applications, C=C bonds in the polydienes need to be precisely controlled to achieve a suitable saturation level via hydrogenation and/or be used for further chemical modification such as cross-linking or functionalization (e.g., epoxidation, halogenation, oxidation) to tune chemical, thermal, and mechanical properties of the polymers.

One representative and effective method to control C=C bond content in polydienes is hydrogenation.^20–22^ In general, hydrogenation of polydienes can be achieved via catalytic (e.g., Ziegler–Natta catalyst,^23–25^ Wilkinson catalyst^26,27^ ) or noncatalytic (using *p*-toluenesulfonyl hydrazide (TSH)^28–37^ ) methods. Catalytic hydrogenation methods use metallic catalysts to activate hydrogen molecules and C=C bonds at high hydrogen pressures (temperature up to ∼240 °C and pressure up to ∼20 bar).^23–25^ The catalytic methods require rigorous and laborious setups with high hydrogen pressures with commensurate safety issues. These methods also pose difficulties with high molecular weight polymers because polymer chains need to be adsorbed and diffused through catalyst pores to initiate hydrogenation reactions.^37,38^

On the other hand, a non-catalytic hydrogenation method, such as using diimide to provide hydrogen molecules for C=C bonds, can be accomplished under mild conditions at atmospheric pressure in easily accessible setups.^20,21,28–32,37,38^ Diimide can be generated via thermolysis of *p*-toluenesulfonyl hydrazide (TSH) in a temperature range of 100∼110 °C (see Figure 1b).^36^ This TSH-based, diimide hydrogenation has been studied for PBD- and PI-containing polydienes to achieve different hydrogenation levels (up to >99.9%) with precision. In general, non-catalytic, TSH-based diimide hydrogenation can offer a safer, more accessible, and easier method of hydrogenation under mild reaction conditions. Thus, in the literature, PBD-containing polydienes have been extensively studied via diimide hydrogenation. However, interestingly, only limited reports are available for the hydrogenation kinetics studies using PI-containing polydienes.

**Figure 1:**
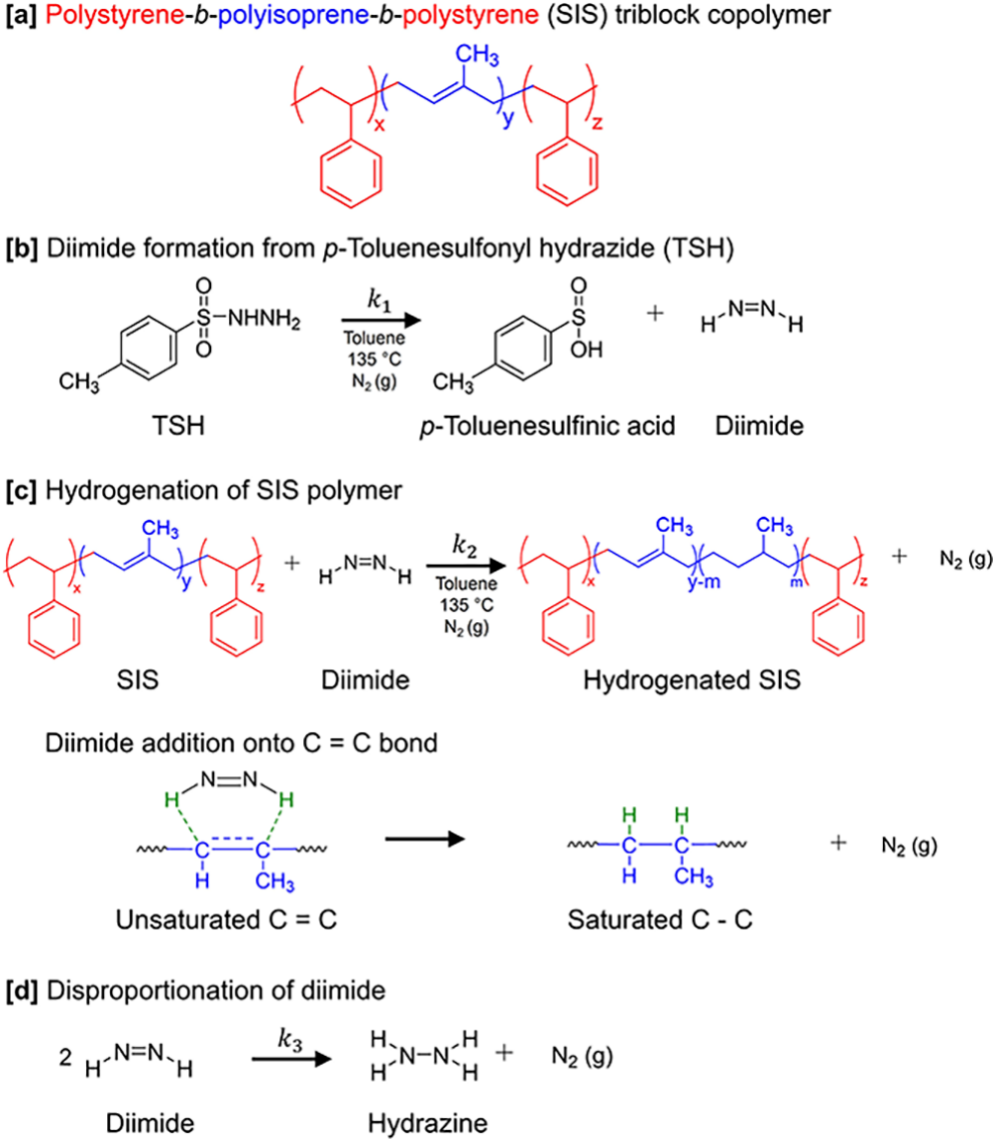
(a) Chemical structure of polystyrene-*b*-polyisoprene-*b*-polystyrene (SIS) triblock copolymer with *x* = *z* = 222 and *y* = 2405 mol of repeating units. (b) Diimide formation from thermolysis of *p*-toluenesulfonyl hydrazide (TSH). (c) Diimide-based hydrogenation of SIS polymer. Diimide provides two hydrogen molecules onto C=C bonds in PI block. (d) Disproportionation of diimide, forming hydrazine and N_2_.

Specifically, polyisoprene (PI)-containing polydienes have gained attraction in various applications due to their mechanical stability and elasticity, versatility to control molecular conformation and microstructure, scalable production, and accessible chemical modification to achieve a desirable structure–property set.^1,14–16,39–42^ In stark contrast to PBD-containing polydienes which largely generate semicrystalline structures after hydrogenation, the presence of a methyl (−CH_3_) group attached to a C=C bond in *cis*-1,4-PI-containing polydienes can hinder crystalline formation, leading to various non-crystalline morphologies.^1,15^ These available non-crystalline morphologies make PI-containing polymers advantageous for broader polymer applications. Also, cross-linking densities can be tuned via controlling C=C bond content, while remaining C=C bonds can be further functionalized, providing a useful toolbox for tuning polymer properties in these materials.

In particular, by combining the versatile benefits of PI-based polydienes with block copolymer architecture, our ultimate goals are to develop the design principles of polymer membranes using this platform, and thus, to build a systematic library of polymer membranes for membrane-based applications. Toward this goal, we selected one representative PI-containing block copolymer, i.e., polystyrene-*b*-polyisoprene-*b*-polystyrene (SIS) triblock copolymer as our initial model polymer platform to generate polymer membranes with different function, structure and transport properties. The SIS block polymer is a commercially available, thermoplastic elastomeric polydiene. It is relevant to broader polymer applications including adhesives, coatings, packaging, blends, industrial components, and polymer membranes due to its mechanical stability, scalable production, and easier chemical modification.^4–11^ In this polymer platform, first, C=C bonds in the PI block can be used for cross-linking and/or chemical functionalization after hydrogenation to obtain a desirable morphology and properties.^3,12,13,42^ Second, the PS blocks can also be separately functionalized (e.g., sulfonation, acetylation, benzoylation) to form ion or water permeating domains. Third, relevant polymer morphologies can be achieved via controlling its block composition in the SIS polymer. Triblock copolymer architecture was chosen because of its mechanical stabilities. Overall, the versatility to control functionality, morphology and transport properties in a systematic manner is the major motivation to select the SIS polymer as our model polymer platform to demonstrate the proof-of-concept.

As a starting point to develop a systematic library of SIS–based polymer membranes, it is important to precisely control the amount of unsaturated C=C bonds in the PI block for further chemical modifications. To achieve this goal, hydrogenation kinetics in SIS polymers need to be understood to correlate reaction conditions with resulting hydrogenation levels. In the literature, PBD-containing polymers have been extensively studied via diimide hydrogenation including both hydrogenation kinetics and single data sets of hydrogenation (see Section 3.3). However, kinetics studies of PI-containing polymers via diimide hydrogenation are largely underdeveloped. To the best of our knowledge, only one hydrogenation kinetics study using PI homopolymer (M̅_W_ ≈ 300 kg/mol) is available via diimide hydrogenation,^31^ but no kinetics studies describing the hydrogenation of PI-containing copolymers are available. Only single data sets using different polymer architectures, compositions, and hydrogenation conditions are sporadically available, making proper comparison difficult among these reports.

Therefore, in this paper, for the first time, we reported a systematic hydrogenation kinetics study of one representative PI-containing polymer, i.e., polystyrene-*b*-polyisoprene-*b*-polystyrene (SIS) triblock copolymer with high molecular weight (M̅_W_ = 210 kg/mol) via TSH-based, diimide hydrogenation using NMR spectroscopy, SEC, and thermal analyses. Diimide hydrogenation provides mild reaction conditions where a broad range of hydrogenation levels (HL = 0∼99.9%) can be achieved without degrading other copolymer blocks.^20,28–32,37^ We recorded hydrogenation level (HL = 0∼99.9%) vs reaction time (*t* = 0∼24 h) of the SIS polymer. We then compared our systematic kinetics results with other PBD- and PI-containing polymers with different chain architectures (e.g., homopolymer, random, or block copolymers), compositions (e.g., PI or PBD contents, copolymer identities), and hydrogenation reaction conditions (e.g., temperature, reaction time, and TSH-to-polymer ratio) in the literature, to evaluate universal efficacy of the diimide hydrogenation method and to build a comprehensive understanding among these reports. We also correlated the hydrogenation level vs thermal decomposition temperature (*T*_d_) and glass transition temperature (*T*_g_) in the SIS polymers, since thermal properties and glass transition are sensitive to chain motion and polymer microstructure.

## 2 Materials and Experimental

### 2.1 Materials

In this study, polystyrene-*b*-polyisoprene-*b*-polystyrene (PS–PI–PS or SIS) triblock copolymer (432415, MilliporeSigma, Burlington, MA) with a weight-average molecular weight, M̅_W_ = 210 kg/mol and a vol % of the PS block = 20% (PS–PI–PS = 23k–164k–23k) was used as shown in Figure 1a. The SIS triblock copolymer is synthesized by anionic polymerization, and its dispersity (Đ) is 1.01, consistent with the previous reports.^43–45^ The SIS polymer contains about 94% 1,4-polyisoprene (and ∼6% 3,4-polyisoprene) as confirmed by ^1^H NMR.^46^ The block composition and density of the SIS polymer are summarized in Table 1. Our measured density value (0.921 g/mL) matches with the reported density (0.93 g/mL) from the manufacturer. The SIS polymer was purchased and used as received.

**Table 1:** SIS Polymer in This Study

M̅_W_ ^a^ [g/mol]	M̅_W_ of PS block [g/mol]	M̅_W_ of PI block [g/mol]	vol % of PS block	vol % of PI block	density [g/mL]	measured density^b^ [g/mL]
210,000	46,200	163,800	20	80	0.93	0.921 ± 0.007

a Reported by the manufacturer.

b Measured at room temperature using a density measurement kit via the Archimedes’ principle. Isopropyl alcohol was used as an auxiliary solvent.

### 2.2 Hydrogenation of SIS Polymer

C=C bonds in PI block of SIS polymer can be hydrogenated to achieve a different saturation level in the polymer. In this study, *p*-toluenesulfonyl hydrazide (TSH) (97%, 132004, MilliporeSigma, Burlington, MA) was selected as a hydrogenation reactant, due to its mild reaction condition and easier accessibility.^28–30^ Figure 1b illustrates diimide formation from TSH. Figure 1c describes the hydrogenation reaction via diimide and chemical structure of the resultant partially saturated SIS polymer.

Note that, in this study, a tertiary amine was not used in hydrogenation reactions to achieve higher extent of hydrogenation without chain degradation in our SIS polymer. For hydrogenation of PBD-containing polymers, a tertiary amine (e.g., tri-*n*-propylamine (TPA)) has been used to reduce side reactions (e.g., disproportionation of diimide), leading to high extents of hydrogenation. In contrast, for hydrogenation of PI-containing polymers, the extent of hydrogenation has been reported low in the presence of tertiary amines, and severe chain degradation occurred under these conditions.^30,31^ Because of these reasons, researchers performed the hydrogenation of PI-containing polymers without tertiary amines and successfully achieved high hydrogenation levels (HL = 97∼99.9%) without chain degradation. For instance, without using tertiary amines, Harwood et al. achieved a HL = 97% in PI homopolymer,^29^ Phinyocheep et al. reached a HL = 98.4%,^32^ Kaewsaiha et al. showed a HL > 99.9%,^33^ and Steube et al. achieved a HL > 99.9%^34^ using PI–PS copolymers. Higaki et al. showed a HL > 99.9% with PS–PI–PS copolymer.^35^ In our study, without using tertiary amines, we also achieved a HL > 99.9% with no side reactions, as consistent with the previous reports. Details will be discussed in Section 3.1.

In this work, toluene was used as the solvent for hydrogenation to achieve a high extent of hydrogenation and minimize byproduct substitution. These hydrogenation targets require relatively slow thermal decomposition of the TSH. The slow TSH decomposition can be achieved via balancing between the amount of excess TSH (i.e., TSH-to-polymer ratio) and the reaction temperature (>toluene boiling point). Similar observations and reaction conditions have been reported elsewhere^28,34,36^ (Table 3). Thus, we used toluene as the solvent with the TSH-to-polymer ratio of 5.25:1 to provide a favorable reaction environment for complete hydrogenation and minimal side reactions.

**Table 2:** Hydrogenation Level (HL) and Thermal Properties of SIS Polymers^b^

reaction time, *t* [h]	hydrogenation level, HL [%]	*T*_d_ [°C]	*T*_g_ of PI block [°C]	*T*_g_ of PS blocks [°C]
0	0	357 ± 3	–59 ± 1	98 ± 2
1	50.5 ± 3.5	388 ± 2	–59 ± 1	98 ± 1
3	75.8 ± 2.4	411 ± 5	–57 ± 1	99 ± 2
8	94.9 ± 0.5	424 ± 3	–56 ± 1	99 ± 0
24	>99.9^a^	421 ± 5	–56 ± 0	100 ± 3

a An average value with a standard deviation is reported for each set. At least three replicate independent samples were used.

b The peak intensity of unsaturated C=C bonds is very small and could not be detected by NMR (below the noise level). Thus, complete hydrogenation (HL > 99.9%) is assumed.

**Table 3:**
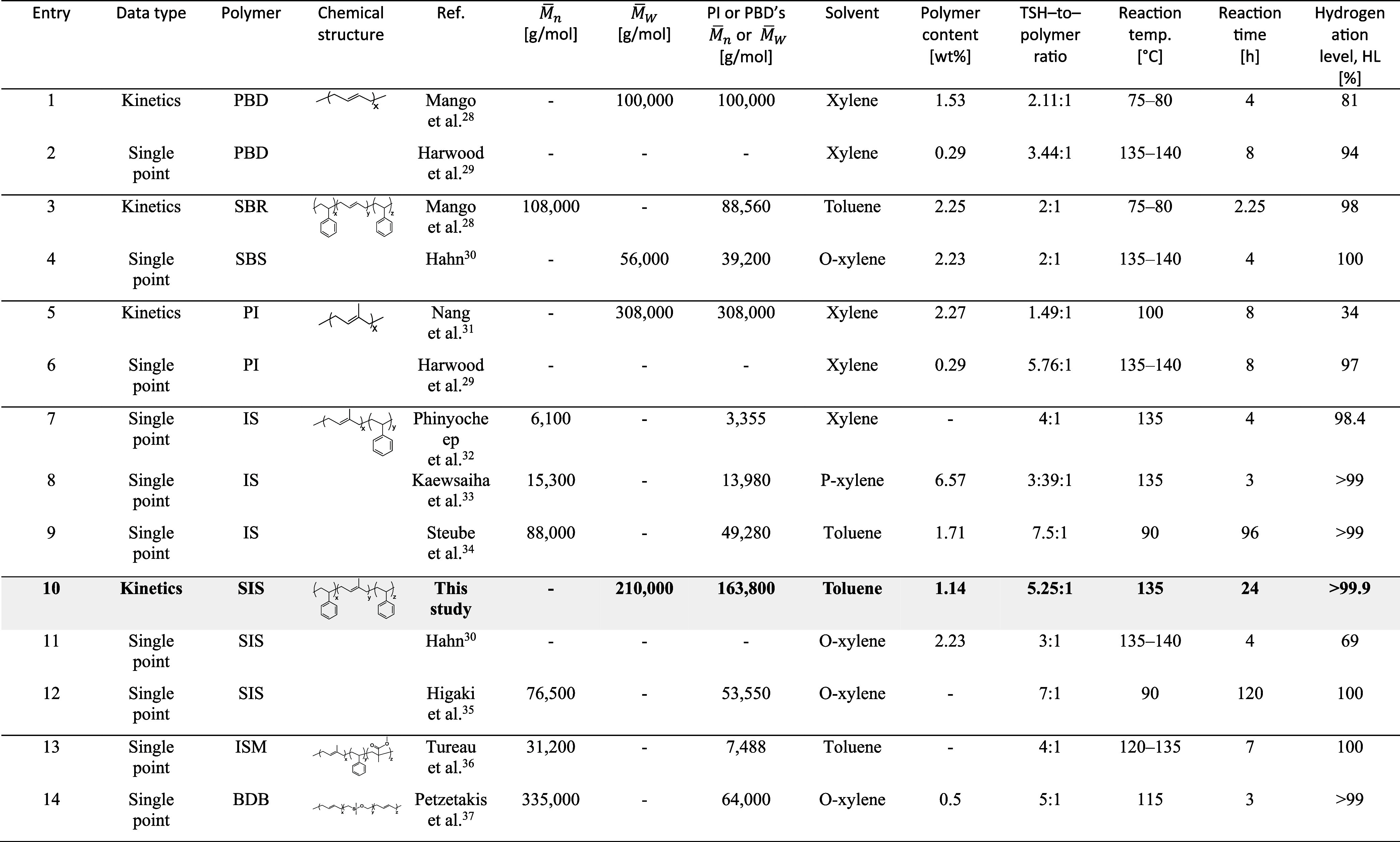
Hydrogenation Reaction Data of SIS Polymers in This Study (Entry 10) and Other PBD- and PI-Containing Polymers in the Literature via TSH-Based, Diimide Hydrogenation

First, 5 g of SIS polymer were dissolved in 200 mL of toluene (≥99.5%, 179418, MilliporeSigma, Burlington, MA) in a three-necked flask on a magnetic stirrer (C-MAG HS 7 digital, IKA, Wilmington, NC) at room temperature (21–22 °C) to form a homogeneous, transparent polymer solution. The SIS polymer solution flask then was immersed in a silicone oil bath (129230, BeanTown Chemical, Hudson, NH) at an elevated temperature (135 °C) for 1 h with a constant N_2_ gas flow (Ultra High Purity 99.999%, UN1066, Linde, Danbury, CT). Second, a separate solution of 56 g TSH in 300 mL toluene was prepared and well-mixed on a magnetic stirrer in a silicone oil bath at 90 °C for 1 h to obtain a homogeneous, transparent solution. Next, the TSH solution was added to the SIS polymer solution flask to start the hydrogenation reaction. An aliquot of 20 mL was collected using a syringe at different reaction times (*t* = 0, 1, 3, 8, 24, and 48 h) to record the hydrogenation level of SIS polymer as a function of reaction time. After the target reaction time was reached, methanol (≥99.8%, 179337, MilliporeSigma, Burlington, MA) was used to terminate the hydrogenation reaction. To precipitate the polymer, 1.5 L of cold methanol was well-mixed with the polymer solution overnight to remove unreacted reactants from the polymer. The precipitated polymer was vacuum filtered using a filter paper (Qualitative Filter Paper grade 413, VWR, Radnor, PA) and then was dissolved in 250 mL of toluene again to form a homogeneous polymer solution. The precipitation procedure with methanol was repeated twice. Subsequent ^1^H NMR analysis confirmed that the unreacted reactants were successfully eliminated (see Figure 2). The purified polymer was then dried in an oven (281A, Thermo Fisher Scientific, Waltham, MA) at 80 °C for 24 h to evaporate solvents. All reactants and solvents were used as received.

**Figure 2:**
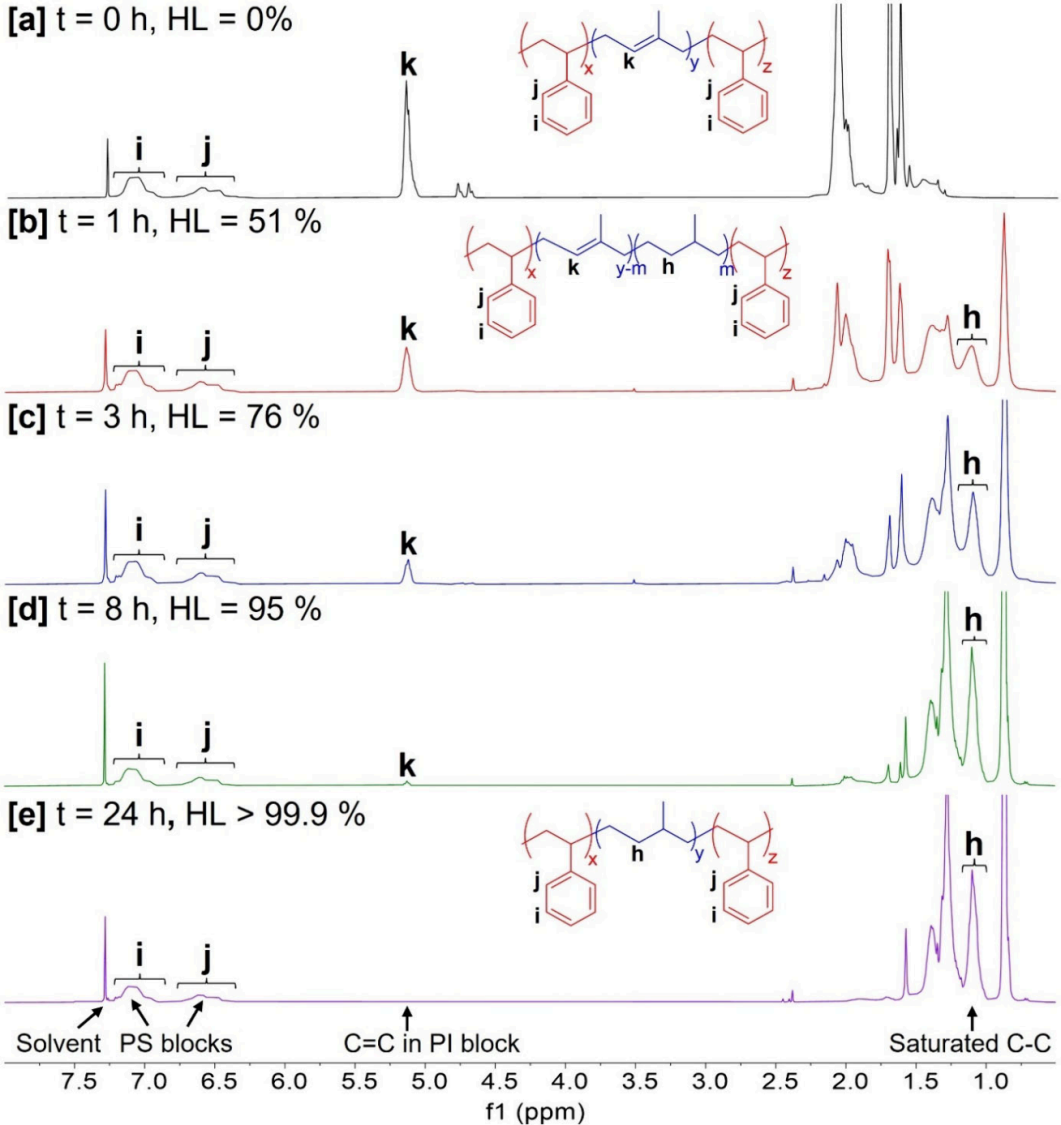
^1^H-NMR spectra of SIS polymers with different hydrogenation levels (HL). Chemical structures with peak assignments are shown. (a) Control, initial SIS polymer with HL = 0% (reaction time, *t* = 0 h). SIS polymers with (b) HL = 51% (*t* = 1 h), (c) HL = 76% (*t* = 3 h), (d) HL = 95% (*t* = 8 h), and (e) HL > 99.9% (*t* = 24 h).

### 2.3 Polymer Characterization

#### 2.3.1 ^1^H-NMR and ^13^C-NMR Spectroscopy

Chemical structure and hydrogenation level of SIS polymers were identified using proton nuclear magnetic resonance spectroscopy (^1^H-NMR). To complement ^1^H NMR analyses, ^13^C-NMR spectroscopy was conducted for detailed peak assignments (see Figures S2–S4). For both ^1^H-NMR and ^13^C-NMR, a polymer sample was dried in a vacuum oven at 50 °C overnight to remove any moisture. Twenty milligrams of the dried polymer sample was dissolved in 1 mL of deuterated chloroform (99.8% CDCl_3_, DLM7100, Cambridge Isotope Laboratories, Andover, MA) with a stir bar at room temperature overnight. Then the transparent, homogeneous polymer solution was transferred to an NMR tube (Wilmad WG-1000, VWR, Radnor, PA) for NMR measurements. ^1^H-NMR spectrum was collected in a 400 MHz ^1^H-NMR instrument (AVANCE NanoBay NEO-400, Bruker, Billerica, MA) at 25 °C with 32 scans and relaxation time of 12 s. ^13^C-NMR spectrum was collected in a 600 MHz ^13^C NMR instrument (AVANCE NEO-600, Bruker, Billerica, MA) at 25 °C with 128 scans and relaxation time of 2 s. The NMR spectra were analyzed using Mnova software (Mestrelab, Santiago de Compostela, Spain).

#### 2.3.2 Size Exclusion Chromatography (SEC)

To measure number average (M̅_n_) and weight average (M̅_W_) molecular weights and polydispersity (Đ) of a polymer, a GPC (Tosoh Bioscience, Tokyo, Japan) equipped with a multiangle light scattering detector (Wyatt Technology, Santa Barbara, CA) and a single column was used with tetrahydrofuran (THF) as the mobile phase. A flow rate of 0.35 mL/min was used for all measurements. Narrow-distribution polystyrene (PS) standards were used to calibrate the system. A polymer solution was prepared at a concentration of 5 mg/mL in THF and passed through a 0.2 μm PTFE filter before measurement. At least three replicate samples were run to confirm data reproducibility in each set. Molecular weights were obtained using a specific refractive index increment (d*n*/d*c*) calculated using the d*n*/d*c* values of PS (0.189 mL/g), PI (0.130 mL/g), and poly­(ethylene-*alt*-propylene) (PEP) (0.079 mL/g).^41,47–52^ We assumed the volume additivity to estimate the d*n*/d*c* value of a hydrogenated polymer.

#### 2.3.3 Thermogravimetric Analysis (TGA)

Thermal stability and decomposition temperature (*T*_d_) of SIS polymers with different hydrogenation levels were analyzed using thermogravimetric analysis (TGA) (Discover TGA 550, Waters TA Instruments, New Castle, DE). Before TGA measurement, a polymer sample was dried in a vacuum oven at 50 °C under dynamic vacuum (with liquid nitrogen) overnight to remove water in the sample. Then, 5 mg of the dried polymer sample were transferred to a platinum TGA pan (957571.901, Waters TA Instruments, New Castle, DE) and were run in the TGA with a ramp rate of 20 °C/min between 50 and 600 °C under N_2_ gas (Ultra High Purity (UHP) 99.999%, UN1066, Linde, Danbury, CT) with a flow rate of 60 mL/min. Decomposition temperature (*T*_d_) was determined at the temperature where 5% of the initial mass was lost.

#### 2.3.4 Differential Scanning Calorimetry (DSC)

Thermal properties and glass transition temperature (*T*_g_) of SIS polymers with different hydrogenation levels were studied using differential scanning calorimetry (DSC) (Discovery DSC 250, Waters TA Instruments, New Castle, DE). Prior to DSC measurement, a polymer sample was dried in a vacuum oven at 50 °C under dynamic vacuum (with liquid nitrogen) overnight to remove any water molecules in the sample. Next, 10 mg of the dried polymer sample was placed in an aluminum DSC pan (T_zero_, 901683.901, Waters TA Instruments, New Castle, DE) and sealed with an aluminum DSC lid (T_zero_, 901671.901, Waters TA Instruments, New Castle, DE) using a punch (T_zero_ press, Waters TA Instruments, New Castle, DE). The sealed sample was run from −90 to 200 °C for three cycles using a mechanical cooler (RCS90, Waters TA Instruments, New Castle, DE) at a heating rate of 20 °C/min under N_2_ gas purge (Ultra High Purity (UHP) 99.999%, flow rate = 50 mL/min). The thermograms of the three cycles overlay onto each other, confirming data reproducibility. The *T*_g_ of the sample was determined at the midpoint of the glass transition in the third heating cycle.

## 3 Results and Discussion

### 3.1 Effect of Reaction Time on Hydrogenation Level (HL) in SIS Polymer

Unsaturated polyisoprene (PI) blocks in SIS triblock copolymer were hydrogenated using diimide (NH=NH) generated from *p*-toluenesulfonyl hydrazide (TSH) because of its mild reaction conditions and easier accessibility, as shown in Figure 1b,c. To accurately estimate the amount of unsaturated C=C bonds in SIS polymer after hydrogenation, we used ^1^H-NMR and ^13^C-NMR analyses, as shown in Figure 2 and Section S1. The chemical structure and peak assignment of the control, initial SIS polymer (before hydrogenation, HL = 0%) are shown in Figure 2a. The control ^1^H-NMR spectrum shows benzene rings (**i** at δ = 7.1 ppm and **j** at δ = 6.6 ppm) in the PS blocks and C=C bonds (**k** at δ = 5.1 ppm) in the PI block as reported in literature.^13,53^ Peaks around δ = 2.5∼1.5 ppm originate from polymer backbone in SIS polymer. Deuterated chloroform (CDCl_3_) solvent peak is also shown at δ = 7.3 ppm as consistent with previous reports.^53^ Detailed peak assignments were conducted using ^13^C-NMR and are discussed in Section S1.

Figure 2b–e shows ^1^H NMR spectra of hydrogenated SIS polymers with different hydrogenation levels (HL = 50∼99.9%) as a function of reaction time (*t* = 1∼24 h). As reaction time increases from 0 to 24 h, the peak intensity of unsaturated C=C bonds (**k** at δ = 5.1 ppm) in the PI block decreases, while the peak intensity of hydrogenated saturated C–C bonds (**h** at δ = 1.1 ppm) in the PI block increases, respectively. When the reaction time reaches 24 h (see Figure 2e), the peak intensity of unsaturated C=C bonds (**k** at δ = 5.1 ppm) disappears. This coupled change between C=C and C–C bonds in the PI block indicates successful hydrogenation of the PI block. Using the area under the curves at these related peaks, the amount of unsaturated C=C and saturated C–C bonds in the PI block can be estimated.

In principle, the decreased amount of unsaturated C=C bonds in the PI block corresponds to an increased amount of hydrogenated saturated C–C bonds in the PI block. Also, the total amount of unsaturated C=C bonds of the PI block in the initial SIS polymer (HL = 0% in Figure 2a) matches with the total amount of saturated C–C bonds of the PI block in the fully hydrogenated SIS polymer (HL > 99.9% in Figure 2e). Using these reasonable assumptions, the hydrogenation level (HL) of SIS polymers can be estimated as

1 $$\mathrm{Hydrogenation}\;\mathrm{Level}\;\left(\mathrm{HL}\right)\;\left[\%\right]=\frac{\mathrm{Moles}\;\mathrm{of}\;\mathrm{saturated}\;C-C\;\mathrm{in}\;\mathrm{PI}\;\mathrm{block}}{\mathrm{Moles}\;\mathrm{of}\;\mathrm{initial},\mathrm{unsaturated}\;C=C\;\mathrm{in}\;\mathrm{PI}\;\mathrm{block}}\times 100$$

Note that, during hydrogenation, the peak location and intensity of the PS blocks (**i** at δ = 7.1 ppm and **j** at δ = 6.6 ppm) in ^1^H-NMR spectra remain the same as expected. This confirms that our hydrogenation conditions neither change nor degrade the PS blocks. All ^1^H-NMR spectra of SIS polymers at different reaction times are shown separately in Figure S1. To complement ^1^H-NMR, ^13^C-NMR spectra of initial SIS polymer (HL = 0%) and fully hydrogenated SIS polymer (HL > 99.9%) are shown in Figures S2–S4 and discussed in Section S1.2.

To further confirm the polymer’s structural integrity during hydrogenation, we measured molecular weight distribution or polydispersity (Đ) of hydrogenated polymers using SEC. Figure 3 shows SEC traces of control SIS and hydrogenated polymers (HL ≥ 95%) along with a narrow-distribution PS standard of a similar molecular weight (M̅_n_ = 225 kg/mol). A monomodal and narrow molecular weight distribution (Đ ≈ 1.01–1.06) is maintained throughout hydrogenation. The slight peak shift of hydrogenated polymers to the lower elution time upon higher HL originates from the small increase in hydrodynamic volume of hydrogenated polymers, as PI block becomes saturated with increasing HL. Similar observations have been reported by other researchers.^4,5,16,34,39^ Figure S5 further shows SEC traces and Đ values of SIS and hydrogenated polymers with PS standards of different molecular weights. Both NMR and SEC results support that our mild diimide hydrogenation conditions preserve the polymer integrity and do not lead to degradation, cross-linking or changes in molecular weight distribution up to 48 h.

**Figure 3:**
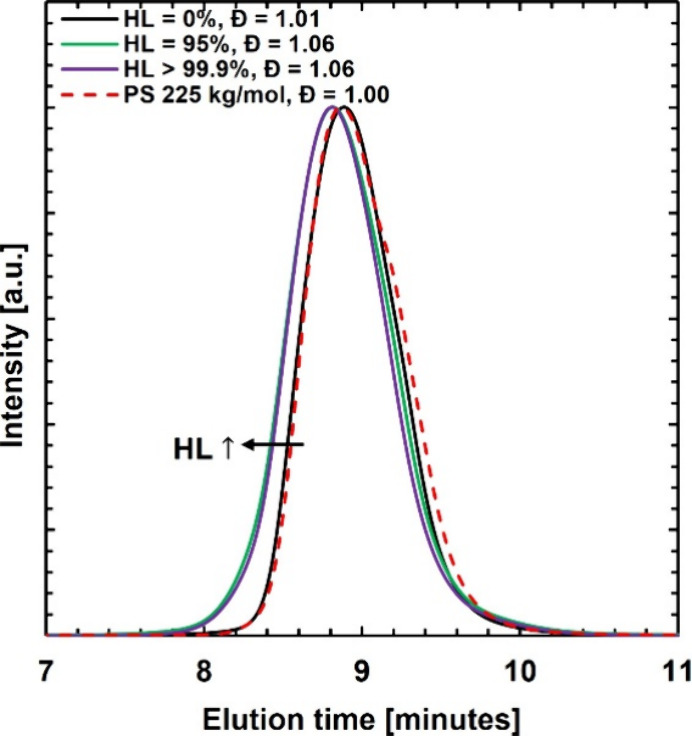
SEC trace of control SIS (HL = 0%) and hydrogenated polymers (HL ≥ 95%). A narrow-distribution PS standard (M̅_n_ = 225 kg/mol) is shown as a reference.

No cross-linking occurred during the hydrogenation and subsequent purification processes. After the hydrogenation, transparent, uniform-thickness thin films of SIS polymers with different HLs were successfully prepared via the solution cast method (see Figure S6 in Section S2). The solution-cast films were easily redissolved in toluene to form transparent, homogeneous polymer solutions, confirming that no cross-linking occurred. The redissolved polymers show the same chemical structures by NMR analyses. These sequential analyses also confirm that our hydrogenation reactions, purification, and subsequent processing (i.e., film formation) do not induce side reactions, chain degradation, and cross-linking, as also confirmed by other reports.^54^

Figure 4 summarizes the effect of reaction time (*t*, [h]) on the hydrogenation level (HL, [%]) of SIS polymers. As reaction time increases from 0 to 24 h, the HL of SIS polymer increases from HL = 0% to HL > 99.9%. A rapid hydrogenation of C=C bonds occurs at initial reaction time (1 h reaction time reaches HL = 51%) and hydrogenation reaction slows down to reach a plateau at around 8 h. At a reaction time of 24 h, the peak intensity of unsaturated C=C bonds is very small and could not be detected by the sensitive NMR,^53^ indicating complete hydrogenation (HL > 99.9%). To further confirm complete hydrogenation, we ran a hydrogenation reaction for 48 h. The resultant ^1^H-NMR spectrum after a reaction time of 48 h is identical to that of the reaction time at 24 h as shown in Figure S1, indicating complete hydrogenation. NMR and SEC analyses also confirm that no side reactions occur up to 48 h without affecting other copolymer blocks.

**Figure 4:**
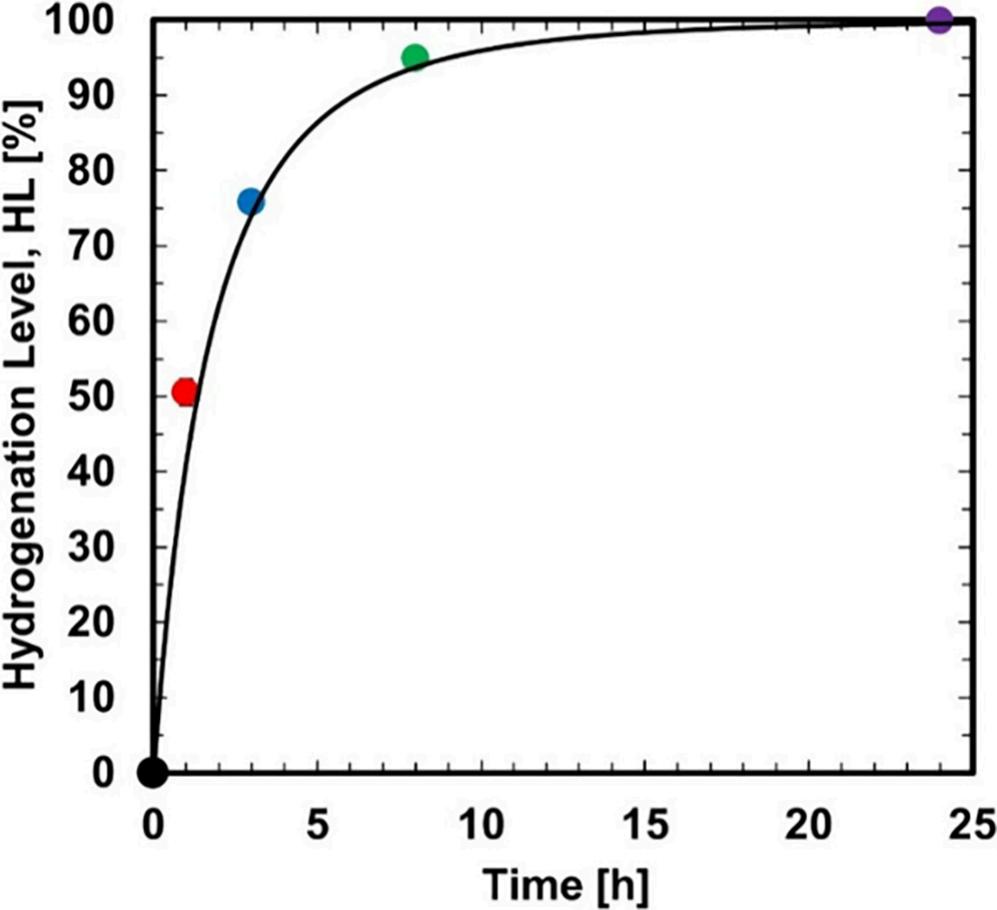
Effect of hydrogenation reaction time on hydrogenation level (HL) of SIS polymers. Symbols were experimental data. The solid line was the best fit using the reaction rates (eqs 2–5) in hydrogenation. Error bars are included.

Hydrogenation of PI block in SIS polymer occurs through three simultaneous reactions including (1) diimide formation from TSH thermolysis (see Figure 1b), (2) diimide hydrogenation of C=C bonds (see Figure 1c), and (3) diimide disproportionation (see Figure 1d). First, diimide, the hydrogenation agent of this study, is generated by thermal decomposition of TSH at elevated temperatures (100∼110 °C).^36^ In this work, diimide formation is a first order reaction (eq 2) and is relatively fast at a high temperature (135 °C).^31^ Next, the formed diimide is consumed by both hydrogenation of C=C bonds in the PI block (eq 3) and disproportionation to form hydrazine (HN–NH) and N_2_ (eq 4).^30,32,37^ In this study, we increased the TSH amount (TSH-to-polymer ratio is 5.25:1, see Table 3) to form excess diimide for the hydrogenation.

2 $$R_{TSH\;thermolysis}=k_{1}\left[TSH\right]$$

3 $$R_{Hydrogenation}=k_{2}\left[C=C\;bonds\;in\;SIS\right]\left[diimide\right]$$

4 $$R_{Disproportionation}=k_{3}{\left[diimide\right]}^{2}$$

where *R* is the reaction rate and *k* is the reaction rate constant. Therefore, the overall change of the diimide concentration during the reaction can be expressed as

5 $$\frac{d\left[diimide\right]}{dt}=k_{1}\left[TSH\right]-k_{2}\left[C=C\;bonds\;in\;SIS\right]\left[diimide\right]-k_{3}{\left[diimide\right]}^{2}$$

The effectiveness of the overall hydrogenation procedure is closely related to the diimide content (d­[diimide]/d*t*) during the reaction.

In this study, our HL vs reaction time data align well with the fitting using the above reaction rates (eqs 2–5) of hydrogenation. The best fit for our experimental data was obtained when *k*_1_ ≫ *k*_2_ > *k*_3_ via the Euler’s method.^55^ This indicates that TSH thermolysis (*k*_1_) is significantly faster at our high temperature range, and thus, diimide is formed immediately after the reaction starts. Also, hydrogenation reaction (*k*_2_) is dominant over diimide disproportionation (*k*_3_), indicating that we can achieve rapid and complete hydrogenation using our reaction conditions. Thus, the initial rapid hydrogenation of SIS polymer (reaction time = 1 h reaches HL = 51%) occurs due to both higher diimide concentration and higher unsaturated C=C bond concentration in the polymer. After 8 h, hydrogenation reaction significantly slows down and reaches a plateau by 24 h due to the decreased amounts of diimide concentration and unsaturated C=C bonds, as expected. Note that error bars in each data set in Figure 4 are very small, indicating precise control of HL vs reaction time in this SIS polymer system. Detailed comparison with other PBD- and PI-containing polymers in the literature will be discussed in Section 3.3.

### 3.2 Effect of Hydrogenation Level (HL) on Thermal Behavior in SIS Polymer

Since thermal behavior such as thermal decomposition and glass transition is sensitive to chain motion and polymer microstructure, we evaluated the influence of HL on thermal decomposition temperature (*T*_d_) and glass transition temperature (*T*_g_) changes in the SIS polymers. First, the effect of HL on thermal decomposition behavior of the SIS polymers is shown in Figure 5. Figure 5a shows weight changes of SIS polymers vs temperature (100∼600 °C), and Figure 5b summarizes HL vs *T*_d_ of the SIS polymers (see Table 2). For comparison, the *T*_d_ values of PI homopolymer (M̅_W_ ≈ 800 kg/mol, *T*_d_ ≈ 350 °C)^54,56–58^ and PS homopolymer (M̅_n_ ≈ 50.1 kg/mol, *T*_d_ ≈ 392 °C)^59,60^ with comparable block sizes of our SIS polymer are shown. The *T*_d_ value of our SIS polymer before hydrogenation (HL = 0%) is comparable to those of PI homopolymer, as expected.

**Figure 5:**
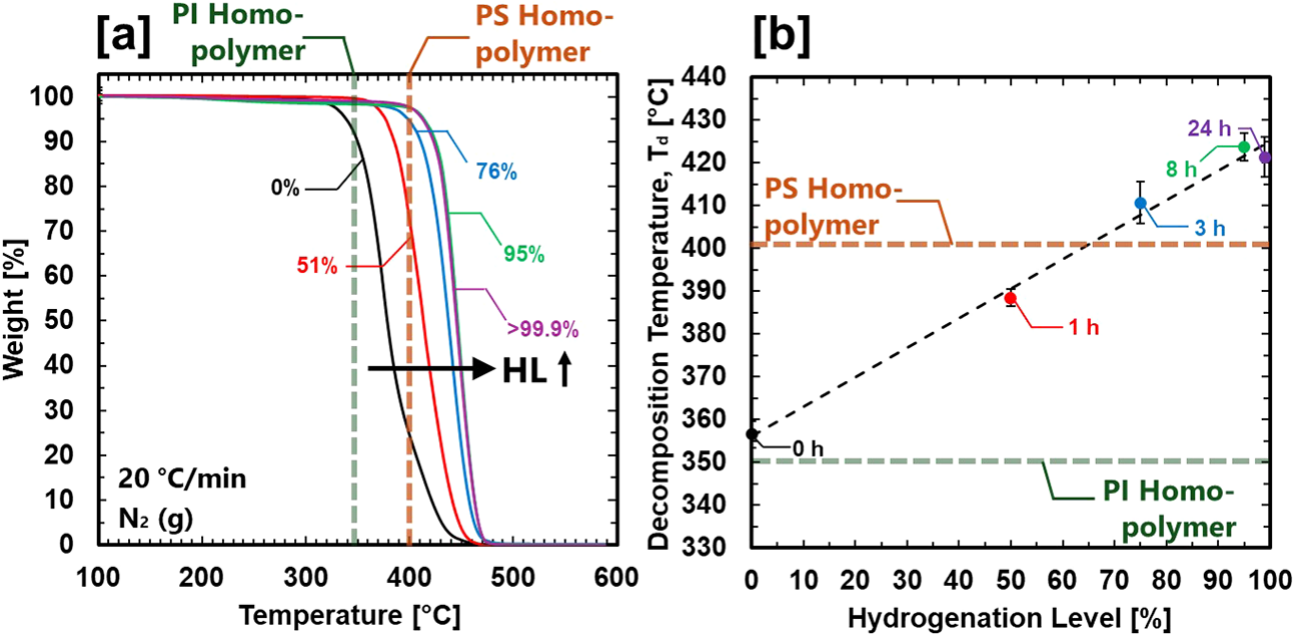
(a) Weight changes of SIS polymers with different HLs vs temperature. *T*_d_ values of PI homopolymer^54,56–58^ and PS homopolymer^59,60^ are shown for comparison. (b) *T*_d_ of SIS polymers vs HL. Sample was heated from 50 to 600 °C, at a rate of 20 °C/min under N_2_ (g) purge. Error bars are included.

In Figure 5a, the overall TGA thermograms of SIS polymers with different HLs exhibit a similar thermal degradation profile, but the onset temperature of thermal degradation is shifted to higher temperatures with increasing HL. As HL increases from HL = 0% to HL > 99.9%, *T*_d_ increases from *T*_d_ = 357 °C to *T*_d_ = 421 °C as shown in Figure 5b. This is due to the increased thermal stabilities of the saturated C–C bonds in the PI block, as reported elsewhere.^1,14,38^ Alkenes with unsaturated C=C bonds are more reactive than alkanes with saturated C–C bonds because of a weaker π bond, which leads to higher chemical reactivity and weaker thermal stability. Weaker thermal stability corresponds to a lower *T*_d_ value.

Second, the effect of HL on thermal behavior and *T*_g_ of SIS polymers is shown in Figure 6. For reference, the *T*_g_s of PI homopolymer (M̅_n_ ≈ 74.7 kg/mol, *T*_g_ ≈ −65 °C)^28,61–63^ and PS homopolymer (PS molecular weight ≈ 50 kg/mol, *T*_g_ ≈ 100 °C)^62,64^ with comparable block sizes of our SIS polymer are also shown.^40,61,64^ Note that the *T*_g_ values of PI homopolymers remains almost constant (−70 to −65 °C) as their molecular weight increases above 2 kg/mol.^40,61,62^

**Figure 6:**
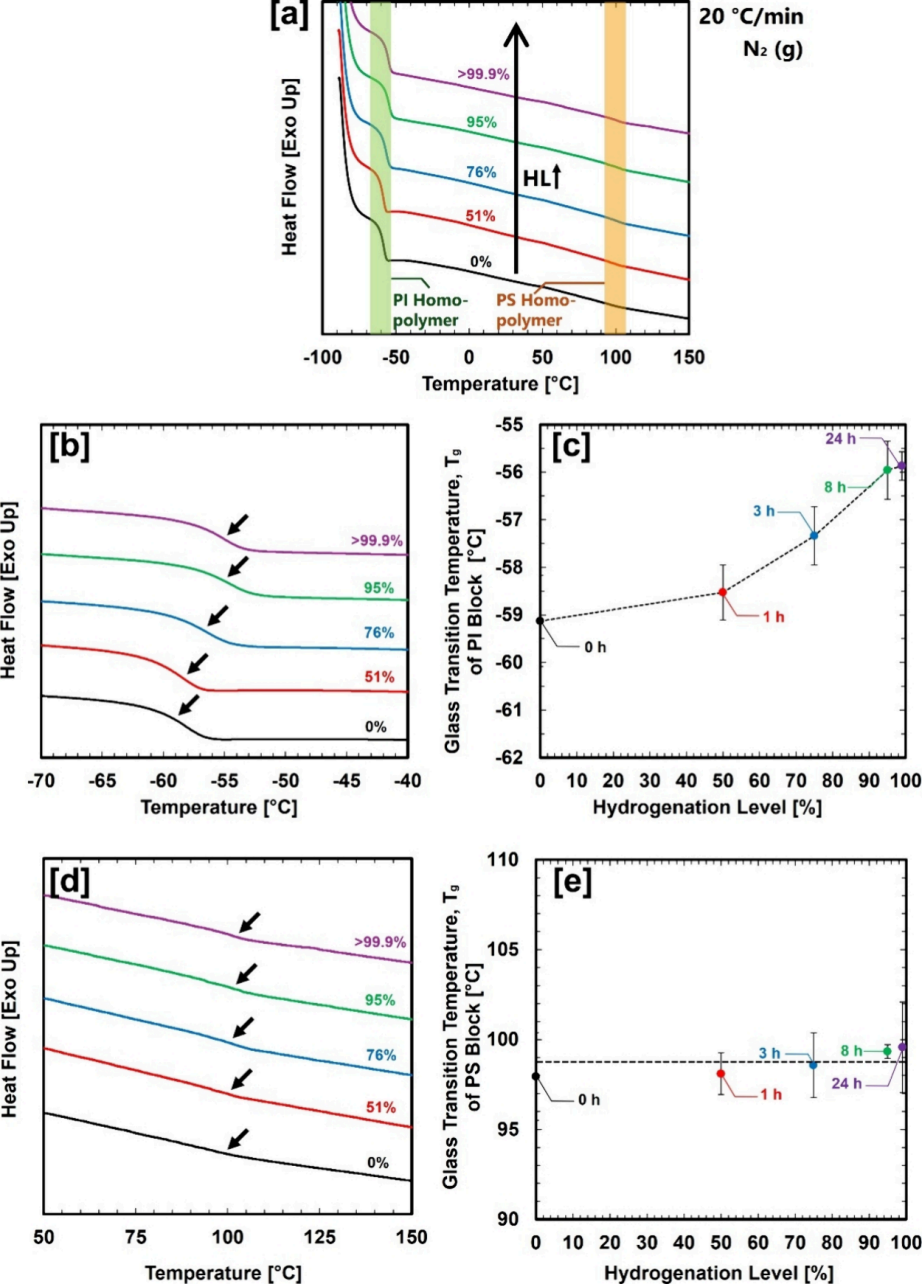
(a) Third scan DSC thermograms of SIS polymers with different HLs vs temperature. Sample was heated from −90 to 200 °C, at a rate of 20 °C/min under N_2_ (g) purge. Thermograms were vertically displaced for easier viewing. *T*_g_ values of PI homopolymer^61,62^ and PS homopolymer^62,64^ are shown for comparison. (b) Magnified thermograms of SIS polymers at PI block’s *T*_g_ range (−59 to −56 °C). Arrows denote *T*_g_ values. (c) *T*_g_ of PI block vs HL. (d) Magnified thermograms of SIS polymers at PS block’s *T*_g_ range (∼99 °C). (e) *T*_g_ of PS blocks vs HL. Error bars are included in (c) and (e).

In Figure 6a, overall DSC thermograms of SIS polymers with different HLs show a similar thermal behavior vs temperature, displaying two *T*_g_ values from the PI and PS blocks, as expected. These two *T*_g_ values are in similar ranges as those of comparable PI and PS homopolymers. Figure 6b shows magnified thermograms focused on the PI block’s *T*_g_ range (−59 to −56 °C) whereas Figure 6d exhibits magnified thermograms at the PS block’s *T*_g_ range (∼99 °C). The PI block’s *T*_g_ transition is more significant than the subtle *T*_g_ transition in the PS blocks, due to the larger volume fraction of the PI block (∼80 vol %) in the SIS polymer.

First, as HL increases from HL = 0% to HL = 76%, the PI block’s *T*_g_ slightly increases from −59 to −57 °C (see Figure 6c). This is due to the competing effects originating from more flexible packing of saturated C–C bonds in the PI block. As HL increases, stiffer unsaturated C=C bonds decrease and more flexible C–C bonds increase, leading to a lower *T*_g_. On the other hand, the saturated C–C bonds enable more effective and closer packing, in contrast to the rigid unsaturated C=C bonds which prevent efficient packing. The increased intermolecular force arising from the increased contact area by more flexible packing of C–C bonds leads to a higher *T*_g_. In our SIS polymers, these competing effects almost offset each other, resulting in a slight *T*_g_ increase. This indicates that the increased intermolecular force originating from the efficient packing of flexible C–C bonds is the dominant factor for slightly increased *T*_g_ in our system.

Similar increasing trends in *T*_g_ have been observed in other hydrogenated polydienes, although the increasing extent of *T*_g_ depends on their chemical structure and conformation.^2,65–69^ For instance, using natural rubber latex (1,4-polyisoprene), as HL increases from HL = 0% to HL = 96% via diimide hydrogenation, its *T*_g_ increases from −64 to −58 °C.^69^ Similarly, natural rubber (polyisoprene) shows increased *T*_g_ from −66 to −43 °C as HL increases from HL = 0% to HL = 87%.^70^

The increasing *T*_g_ vs HL reports are more available with PBD-containing polymers. Hahn showed that *T*_g_ of SBS (PS–PBD–PS) triblock copolymer increases from −62 to −55 °C as HL increases from HL = 0% to HL > 99.9%.^30^ PBD (*cis*-1,4-polybutadiene) homopolymer shows the increased *T*_g_ from −109 to −95 °C with increasing HL from HL = 0% and HL = 61%.^65^ PS–PBD copolymer exhibits the increased *T*_g_ from −51 to −15 °C as HL increases from HL = 0% to HL = 55%.^22^

As HL approaches completion from HL = 76% to HL > 99.9%, the PI block’s *T*_g_ values remain almost the same. Also, the PS block’s *T*_g_ values remain almost the same vs HL, as expected (see Figure 6e). The DSC results, as consistent with our NMR analyses, confirm that our hydrogenation conditions neither affect nor degrade the PS blocks in this polymer.

### 3.3 Comparison with Hydrogenation Results of Other PBD- and PI-Containing Polymers in the Literature

In this section, we compared our hydrogenation kinetics study of the SIS polymer with hydrogenation results of other PBD- and PI-containing polymers via TSH-based diimide hydrogenation in literature. Our motivation was to investigate the universal efficacy of our hydrogenation method and build a comprehensive understanding among these reports, as shown in Figure 7. Other PBD- and PI-containing polymers with (1) different chain architectures (e.g., homopolymer, random copolymer, diblock or triblock copolymers), (2) different molecular weights (e.g., PBD or PI contents varied between 3 and 300 kg/mol), and (3) different copolymer identities (e.g., PS, poly­(methyl methacrylate) (PMMA) or poly­(dimethylsiloxane) (PDMS)), which were hydrogenated via diimide using (4) different hydrogenation reaction conditions (e.g., temperature, reaction time, or TSH-to-polymer ratio), were selected as shown in Table 3. Although polymer identities and detailed hydrogenation conditions were different in these other polymers, these reports were chosen to reasonably compare the effects of (1) chain architectures, (2) C=C bond contents (i.e., PBD or PI contents), (3) copolymer identities, and (4) TSH-based hydrogenation conditions on the resulting HL in the polymers. Thus, for effective comparison, the HL vs reaction time (*t*) data of these other polymers in the literature are plotted along with our kinetics results in Figure 7.

**Figure 7:**
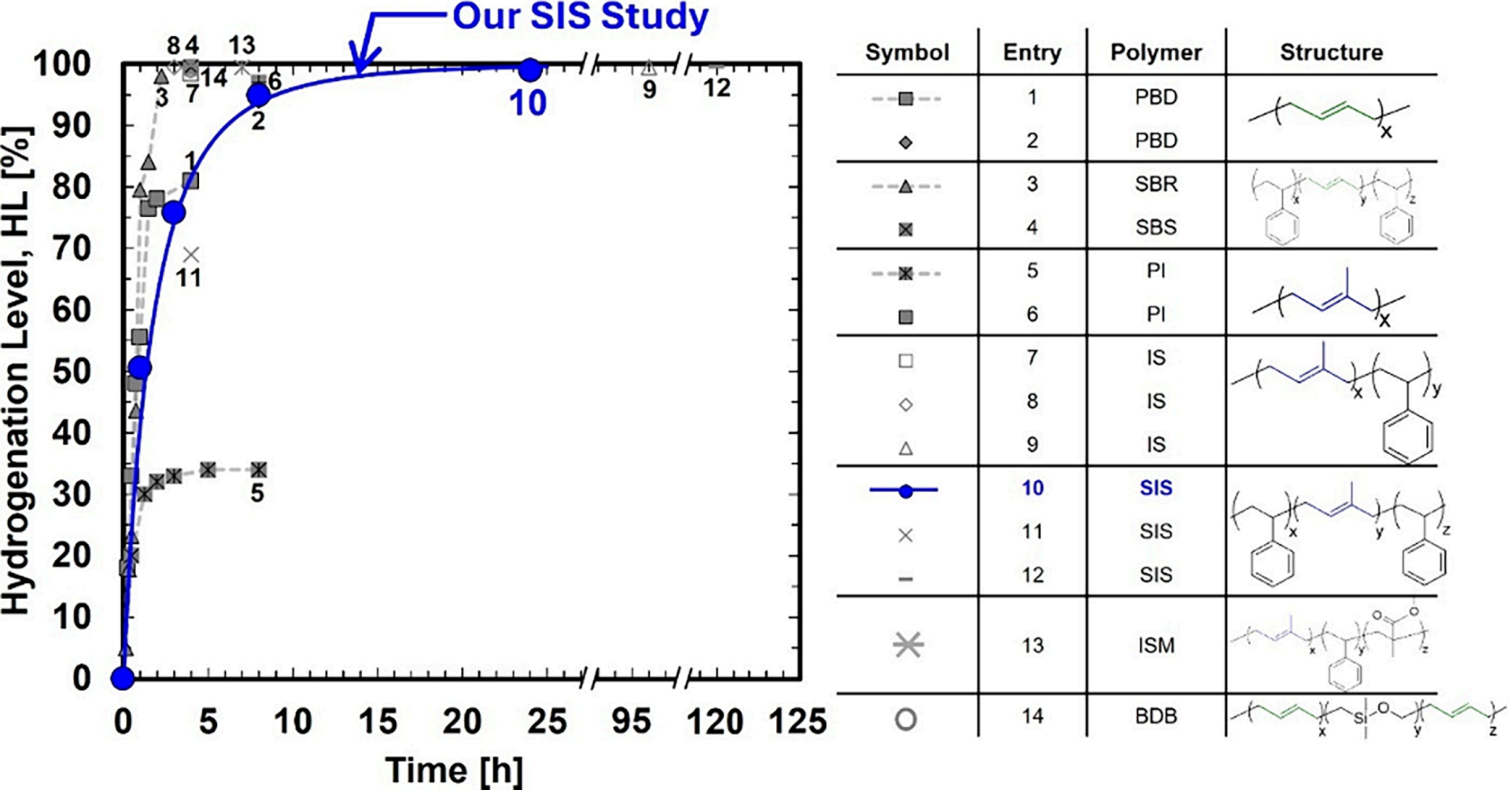
Hydrogenation level (HL) vs reaction time of SIS polymers in this study (Entry 10) and other PBD- and PI-containing polymers in the literature via TSH-based, diimide hydrogenation. Kinetics data are shown with a dashed line and symbols whereas single data sets are shown only with symbols. In the table, the PBD block is shown in green and the PI block is shown in blue for easier viewing.

First, since PBD-containing polymers have been widely studied for hydrogenation in literature, PBD-containing polymers with different chain architectures (e.g., homopolymer, random copolymer, and triblock copolymer) and different TSH-based hydrogenation conditions are compared. Starting with PBD homopolymers (M̅_W_ ≈ 100 kg/mol), as a representative example, Mango et al. (Entry 1) reported the HL (0∼81%) vs reaction time (0∼4 h).^28^ In their report, as reaction time increases, HL increases up to HL = 81%. Using a higher temperature (∼135 °C) and TSH-to-polymer ratio (∼3.44) for a longer time (∼8 h), a higher HL = 94% of PBD homopolymer was achieved by Harwood et al. (Entry 2).^29^ These studies show that, in PBD homopolymers, different HLs can be systematically obtained by controlling reaction conditions.

Similarly, in PBD-containing copolymers, such as random copolymers (PS–PBD) and triblock copolymers (PS–PBD–PS), hydrogenation reactions can still effectively occur in the presence of other long copolymer blocks. Mango et al. (Entry 3) showed relatively rapid hydrogenation (∼2 h) at relatively low temperatures (∼80 °C) to yield a HL = 98% in random copolymers (PS–PBD).^28^ At a higher temperature (135 °C), Hahn (Entry 4) reported that triblock copolymer (PS–PBD–PS) shows a HL > 99.9% at a reaction time = 4 h.^30^ Relatively rapid hydrogenation rate in PBD-containing polymers is likely due to less steric hindrance in butadiene’s C=C bond, compared to isoprene’s C=C bond (with a bulky −CH_3_ group attached to a C=C bond) in PI-containing polymers.

Second, we correlated our SIS polymer’s kinetics results with other PI-containing polymers’ hydrogenation data with different chain architectures, compositions, and hydrogenation conditions, to evaluate the efficacy of our method. Compared to the extensively studied PBD-containing polymers with diimide hydrogenation, hydrogenation kinetics studies of PI-containing polymers are largely underdeveloped. To the best of our knowledge, only one hydrogenation kinetics study using PI homopolymer (M̅_W_ ≈ 300 kg/mol) is available via diimide hydrogenation,^31^ but no kinetics studies describing the hydrogenation of PI-containing copolymers are available. Most hydrogenation reports using PI-containing polymers show limited single data sets using different polymer architectures, compositions, and hydrogenation conditions (see Table 3).

Starting with PI homopolymers (M̅_W_ ≈ 300 kg/mol), Nang et al. (Entry 5) reported relatively lower HL (0∼34%) vs reaction time (0∼8 h).^31^ Lower HL is due to a lower TSH-to-polymer ratio (∼1.49, indicating the lower diimide concentration during hydrogenation) (note that this report used the lowest TSH-to-polymer ratio in Table 3) and a lower temperature range (∼100 °C). In contrast, Harwood et al. (Entry 6) achieved a much higher HL = 97% of PI homopolymer using a higher TSH-to-polymer ratio (∼5.76) at a higher temperature (∼135 °C) in the same reaction time (8 h), as expected for rigorous hydrogenation conditions.^29^

In PI-containing copolymers, such as diblock or triblock copolymers with additional copolymer blocks (e.g., PS, PMMA, or PDMS), hydrogenation reactions nevertheless occur in a similar manner. In diblock copolymer architecture (PS–PI), a higher temperature (∼135 °C) for a reaction time = 3∼4 h yields a HL ≈ 99%, regardless of the PI block size (PI M̅_n_ ≈ 3–11 kg/mol) as reported by Phinyocheep et al. (Entry 7) and Kaewsaiha et al. (Entry 8).^32,33^ In contrast, a lower temperature (∼90 °C) for an extended reaction time (∼96 h) using a similar diblock copolymer (PI M̅_n_ ≈ 49 kg/mol) results in a comparable HL > 99%, as reported by Steube et al. (Entry 9).^34^ These reports again indicate that both temperature range and reaction time are coupled for effective hydrogenation, regardless of chain architecture and copolymer block length.

In triblock copolymer architecture, such as our SIS polymer in this study (Entry 10), systematic hydrogenation kinetics data using a similar triblock structure like ours are not available in literature. Only limited single data reports can be found. For instance, Hahn (Entry 11)^30^ reported that a higher temperature (∼135 °C) hydrogenation for a shorter reaction time = 4 h using a SIS triblock copolymer (M̅_n_ is not reported) yields an HL = 69%, while Higaki et al. (Entry 12)^35^ showed that a lower temperature (∼90 °C) hydrogenation for an extended reaction time ≈ 120 h using a SIS triblock copolymer (PI M̅_n_ ≈ 54 kg/mol) results in an HL ≈ 100%. These limited reports again confirm that a higher temperature and/or a longer reaction time can yield a higher HL, regardless of chain architectures.

Likewise, in the triblock copolymer architecture, when different copolymer identities (PMMA or PDMS) other than the PS block are added, the diimide hydrogenation method enables similarly effective hydrogenation without affecting the copolymer blocks. With different copolymer blocks such as PMMA (Entry 13, PI M̅_n_ ≈ 7000 g/mol)^36^ or PDMS (Entry 14, PBD M̅_n_ ≈ 64,000 g/mol),^37^ a high HL > 99.9% was obtained using an increased temperature (∼135 C°) and a high TSH-to-polymer ratio (∼4) without degrading the copolymer blocks. These results emphasize the universal efficacy of diimide hydrogenation for different copolymer chemistries.

Overall, the TSH-based diimide hydrogenation method is universal and effective over a broad range of PBD- and PI-containing polymers with different chain architectures and compositions. Diimide hydrogenation provides mild reaction conditions where a broad range of HL (0∼99.9%) can be achieved without degrading other copolymer blocks. In general, a higher temperature with a higher TSH-to-polymer ratio yields a higher HL with a faster reaction rate. Compared to other polymers in the literature, our hydrogenation kinetics study of SIS triblock copolymer shows the systematic record between HL (0∼99.9%) and reaction time (0∼48 h). Our kinetics results align well with other similar polymers in the literature but cover a wider range of reaction times (up to 48 h) than those of other reports. NMR and SEC analyses confirm that no side reactions occur up to 48 h without affecting other copolymer blocks. Our extensive search of the literature confirms that this is the first time a systematic hydrogenation kinetics study of PI-containing copolymer has been reported via TSH-based, diimide hydrogenation and compared with other available diimide hydrogenation studies for a comprehensive review. Using this information, one can achieve a desired HL by controlling reaction time for these materials. The resulting polymers can be additionally modified through cross-linking and/or chemical functionalization to obtain a suitable structure–property set for various applications. Our subsequent paper will use this systematic library of hydrogenated SIS polymers to design a universal polymer membrane platform for membrane-based applications in sustainability.

## 4 Conclusions

Polydienes, such as polybutadiene (PBD)- or polyisoprene (PI)-containing polymers, are of great importance in both conventional and emerging applications in energy, environment, and health. For versatile use of polydienes in various applications, C=C bonds in PBD or PI blocks can be hydrogenated to achieve a desirable saturation level and/or be used for further chemical modification such as cross-linking or functionalization to obtain necessary property sets.

In this study, for the first time, we reported a systematic hydrogenation kinetics study of one representative PI-containing polymer, i.e., polystyrene-*b*-polyisoprene-*b*-polystyrene (SIS) triblock copolymer with high molecular weight (M̅_W_ = 210 kg/mol) via a *p*-toluenesulfonyl hydrazide (TSH)-based, diimide hydrogenation. TSH-based diimide hydrogenation can occur under mild conditions with precision, enabling easier and precise control of hydrogenation level (HL) in PBD- and PI-containing polymers. Using this method, we achieved a HL = 51% at a reaction time = 1 h and a HL > 99.9% at a reaction time = 24 h. Our kinetics results align well with other similar polymers in the literature but cover a wider range of reaction times (up to 48 h) than those of other reports. NMR and SEC analyses confirm that no side reactions occur up to 48 h without affecting other copolymer blocks. With increasing HL, the *T*_d_ is shifted to higher temperatures, due to the increased thermal stabilities of the saturated C–C bonds in the PI block. As the HL increases, the PI block’s *T*_g_ slightly increases due to increased intermolecular force from more flexible packing of saturated C–C bonds in the PI block. In contrast, the PS block’s *T*_g_ remains the same as expected, confirming again that our hydrogenation conditions only affect the PI block.

Overall, the TSH-based diimide hydrogenation method is universal and effective over a broad range of PBD- and PI-containing polymers with different chain architectures and compositions. Diimide hydrogenation provides mild reaction conditions where a broad range of HL (0∼99.9%) can be achieved without degrading other copolymer blocks. A higher temperature with a higher TSH-to-polymer ratio yields a higher HL with a faster reaction rate. To the best of our knowledge, this is the first time a systematic hydrogenation kinetics study of PI-containing copolymer has been reported via TSH-based diimide hydrogenation and compared with other available diimide hydrogenation studies for a comprehensive review. Using this information, one can achieve a desired HL by controlling reaction time for these materials. After hydrogenation, further chemical modification such as cross-linking or functionalization can be performed for various applications.

## Supplementary Material



## Notes

^1^H NMR and ^13^C NMR analyses, SEC results, and polymer film formation (PDF)
