# Supplementary material for: Hydrogenation Kinetics Study: Precise Control of C=C Bonds in Polyisoprene (PI)-Containing Block Copolymers via Diimide Hydrogenation
Source: ACS Appl Polym Mater. 2026 Jun 3;8(11):8832–44. doi: 10.1021/acsapm.6c01388 (PMC13270509; doi:10.1021/acsapm.6c01388)
Supplement: Supplementary file 1 [file ap6c01388_si_001.pdf]

Supplemental Information (SI)

Hydrogenation Kinetics Study: Precise Control of C = C Bonds in Polyisoprene (PI)–Containing Block Copolymers via Diimide Hydrogenation

Luis Felipe Caspari Thiele[1], Yongha Kim[2], Corey A. Roberts[3], Shubhra Goel[1], Andrew L. Zydney[2], Ralph H. Colby[1], Manish S. Kelkar[4], Uwe Beuscher[5], and Hee Jeung Oh[1,2,6,7*]

[1]Department of Materials Science and Engineering, The Pennsylvania State University, University Park, Pennsylvania 16802, United States
[2]Department of Chemical Engineering, The Pennsylvania State University, University Park, Pennsylvania 16802, United States
[3]Department of Chemistry, The University of Kentucky, Lexington, Kentucky 40506, United States
[4]AbbVie, North Chicago, Illinois 60064, United States
[5]W. L. Gore & Associates, Newark, Delaware 19711, United States
[6]Institute of Energy and Environment, The Pennsylvania State University, University Park, Pennsylvania 16802, United States
[7]Advanced Manufacturing and Design, The Pennsylvania State University, University Park, Pennsylvania 16802, United States

Corresponding Author: Prof. Hee Jeung Oh
Department of Chemical Engineering
Department of Materials Science and Engineering (by courtesy)
Institute of Energy and Environment (IEE)
Advanced Manufacturing and Design (AMD)
The Pennsylvania State University
Email: hjoh@psu.edu
Phone: 814-863-9085

S1. NMR Characterization

S1.1 ¹H–NMR analyses

Proton nuclear magnetic resonance (¹H–NMR) spectroscopy was used to identify chemical structure and hydrogenation level (HL) of SIS polymers, as explained in ***Section 2.3.1***. Complete NMR spectra of SIS polymers with different HLs are shown in **Figure S1**. Note that (1) deuterated solvent ($CDCl_3$) peak is shown at $\delta = 7.3$ ppm, (2) polystyrene (PS) blocks' benzene peaks are shown at $\delta = 7.1$ (**i**) and $\delta = 6.6$ ppm (**j**), (3) polyisoprene (PI) block's C = C bonds' peak is shown at $\delta = 5.1$ ppm (**k**) (C = C bonds' peak intensity decreases with increasing HL), and (4) hydrogenated saturated C – C bonds' peak is shown at $\delta = 1.1$ ppm (**h**) (saturated C – C bonds' peak intensity increases with increasing HL). Remaining peaks are assigned to different hydrocarbon combinations (e.g., –CH, $–CH_2$, $–CH_3$) which are present in the polymer backbone of the SIS polymer.

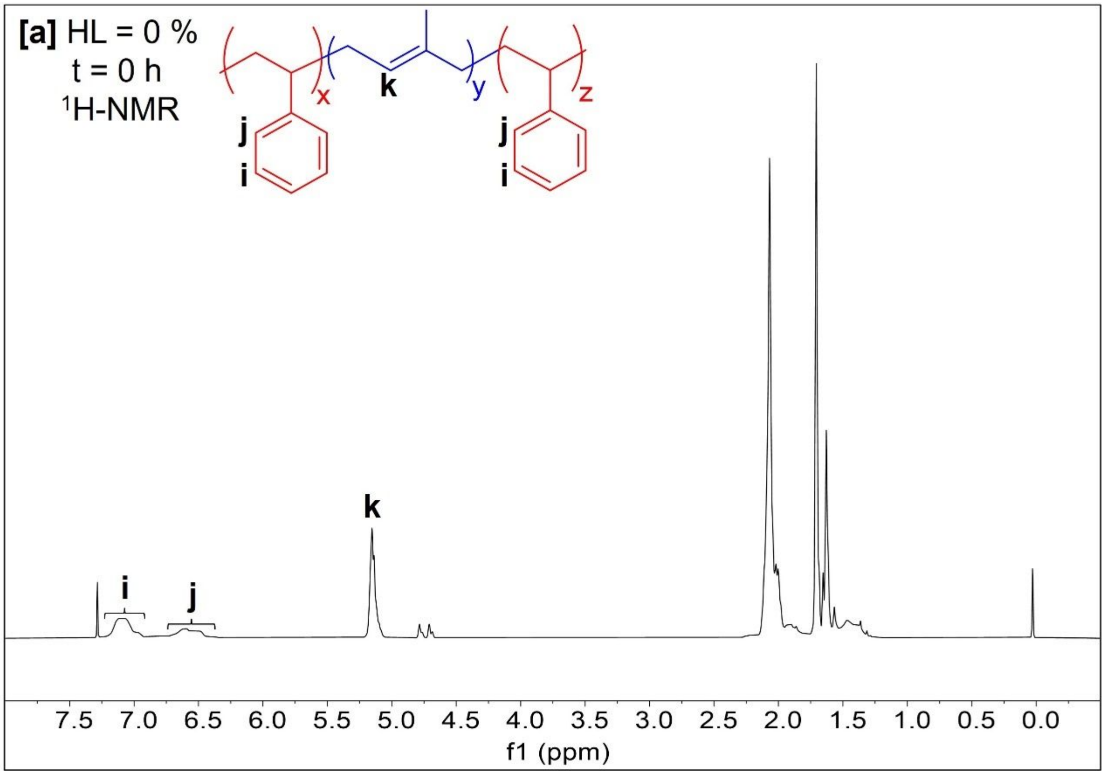

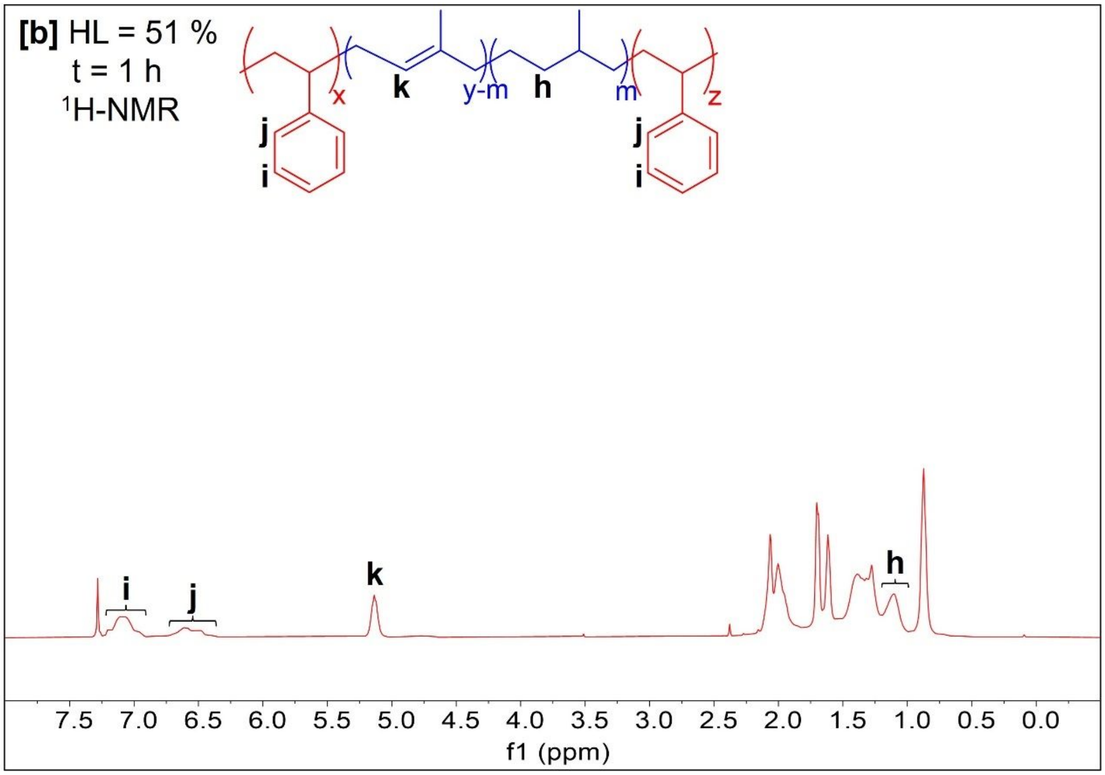

[b] HL = 51 %
t = 1 h
1H-NMR
x
k
y-m
h
m
z
j
i
j
i
k
h
7.5 7.0 6.5 6.0 5.5 5.0 4.5 4.0 3.5 3.0 2.5 2.0 1.5 1.0 0.5 0.0
f1 (ppm)

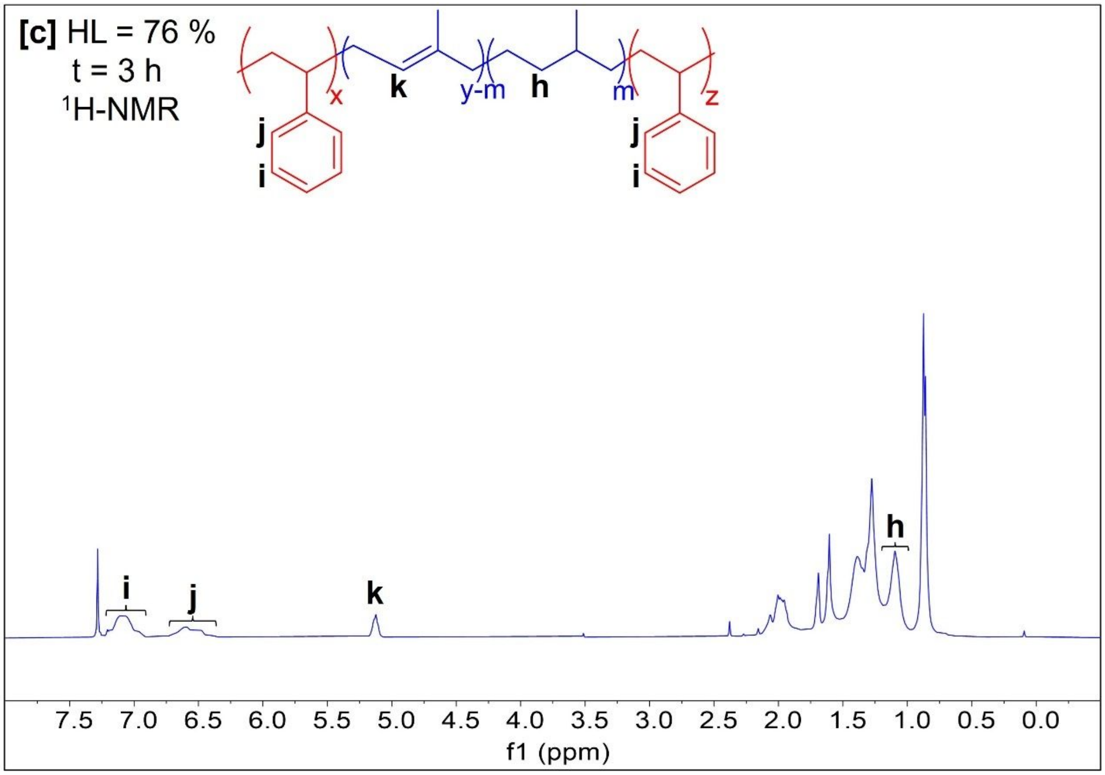

[c] HL = 76 %
t = 3 h
1H-NMR
x
k
y-m
h
m
z
j
i
j
i
k
h
7.5 7.0 6.5 6.0 5.5 5.0 4.5 4.0 3.5 3.0 2.5 2.0 1.5 1.0 0.5 0.0
f1 (ppm)

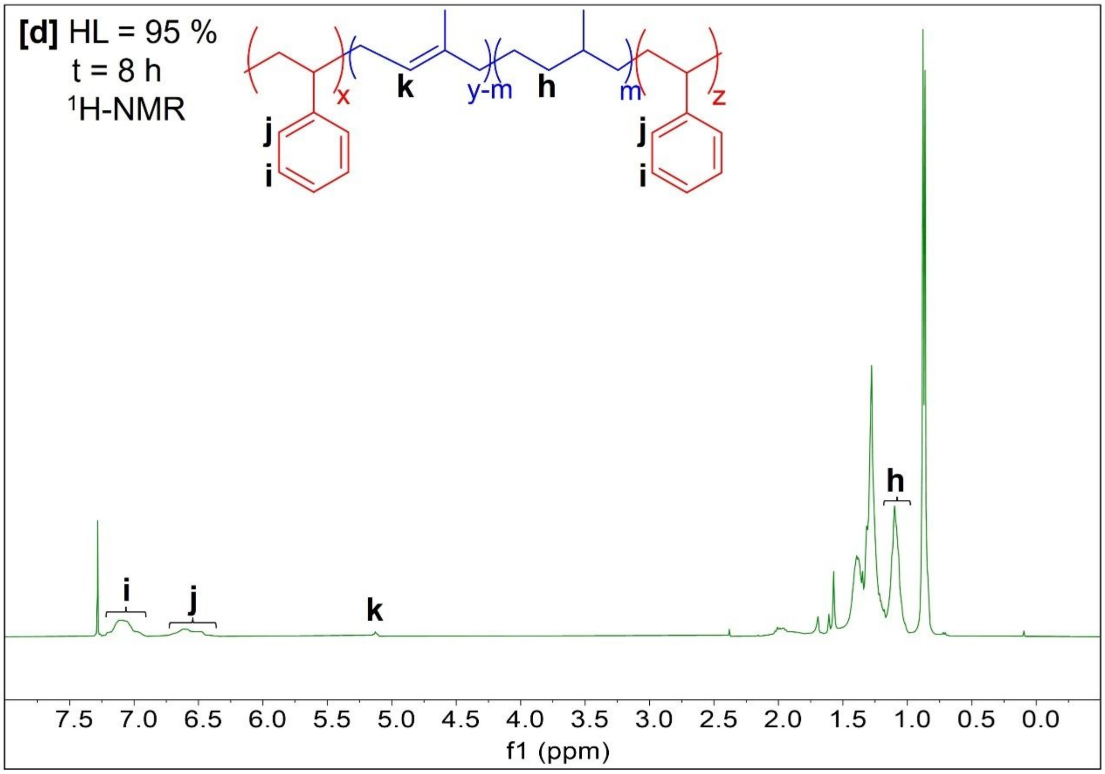
[d] HL = 95 %
t = 8 h
1H-NMR
x
k
y-m
h
m
z
j
i
7.5 7.0 6.5 6.0 5.5 5.0 4.5 4.0 3.5 3.0 2.5 2.0 1.5 1.0 0.5 0.0
f1 (ppm)

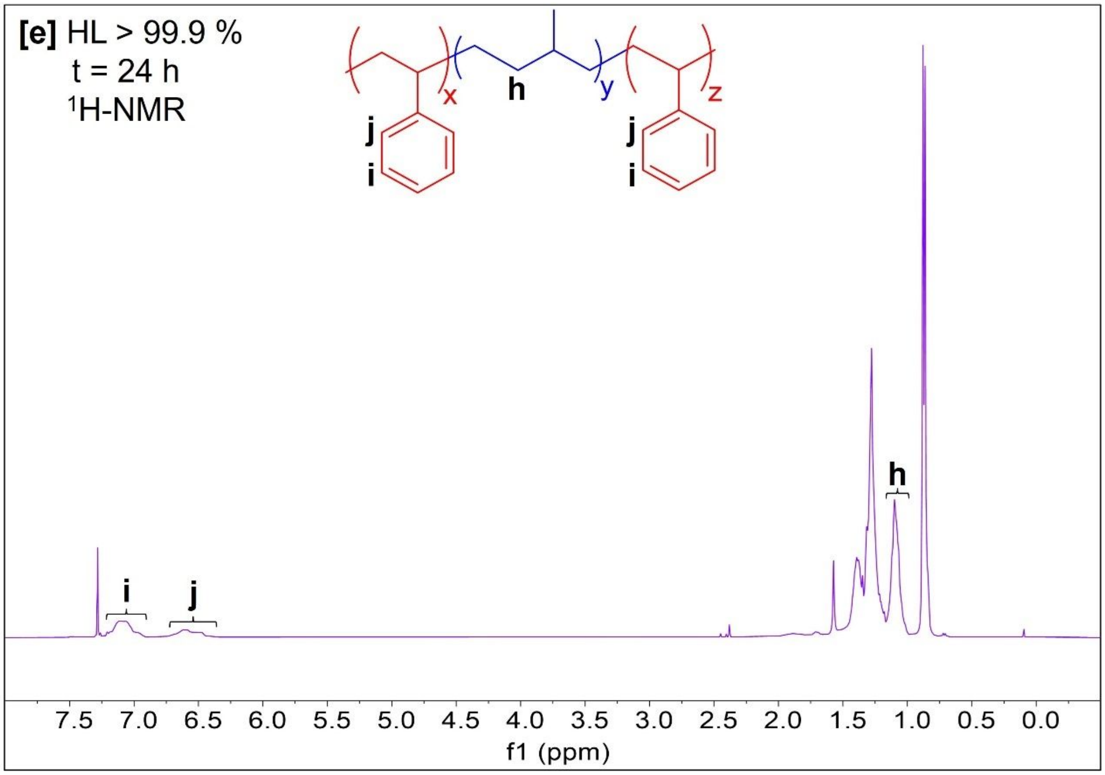
[e] HL > 99.9 %
t = 24 h
1H-NMR
x
h
y
z
j
i
7.5 7.0 6.5 6.0 5.5 5.0 4.5 4.0 3.5 3.0 2.5 2.0 1.5 1.0 0.5 0.0
f1 (ppm)

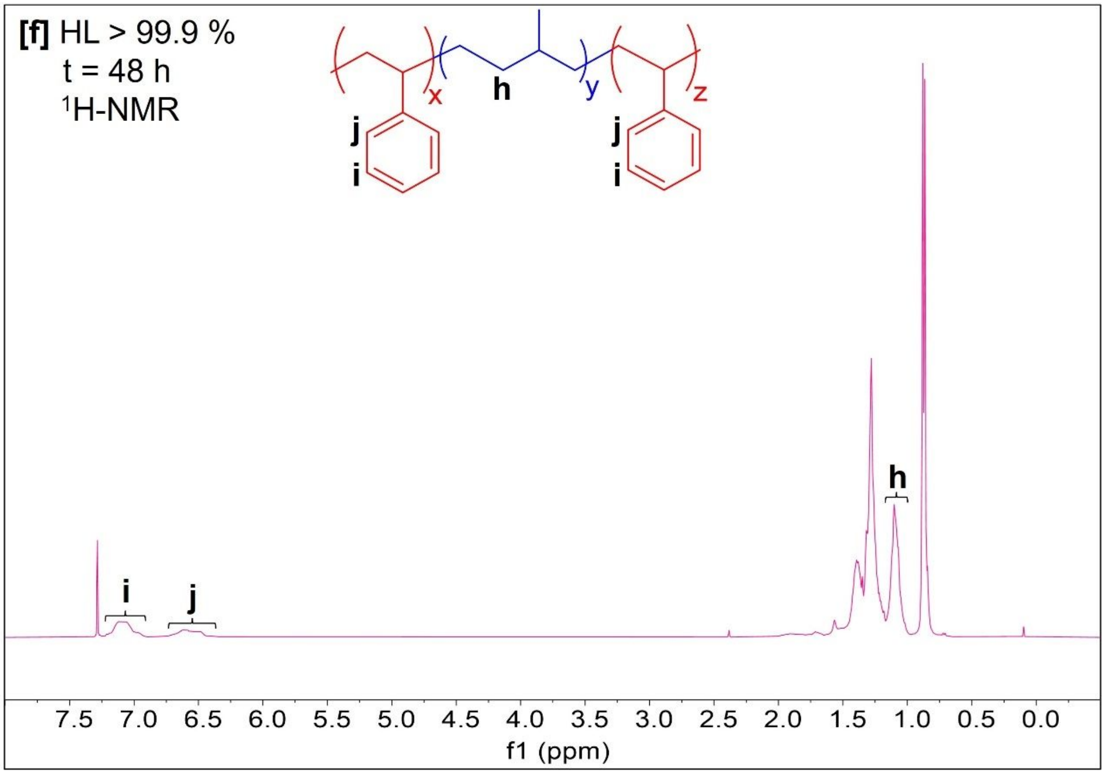

Figure S1. ^{1}H–NMR spectra of SIS polymers with **[a]** hydrogenation level (HL) = 0 % (reaction time, t = 0 h), **[b]** HL = 51 % (t = 1 h), **[c]** HL = 76 % (t = 3 h), **[d]** HL = 95 % (t = 8 h), **[e]** HL > 99.9 % (t = 24 h), and **[f]** HL > 99.9 % (t = 48 h). Chemical structures with peak assignments are shown.

S1.2 ^{13}C–NMR analyses

Carbon nuclear magnetic resonance (^{13}C–NMR) spectroscopy was used to complement ^{1}H–NMR analyses to identify chemical structure of SIS polymers as shown in **Figure S2**. Additionally, we also conducted Heteronuclear Single Quantum Coherence (HSQC) ^{13}C–NMR (see **Figure S3**) and Distortionless Enhancement by Polarization Transfer (DEPT) ^{13}C–NMR (see **Figure S4**) techniques to supplement chemical peak assignments. The DEPT ^{13}C–NMR can distinguish the signals between –CH, $–CH_2$, and $–CH_3$ groups by changing angle parameters, allowing for easier identification and distinction between chemical identities[1-3]. For instance, as shown in **Figure S3**, the positive peaks are originating from either –CH or $–CH_3$ groups, while the negative peaks are correlated to $–CH_2$ groups. The HSQC ^{13}C–NMR can link ^{1}H–NMR and ^{13}C–

NMR spectra together by coordinating chemical shifts[4-6] as shown in **Figure S4**. The combination of these four NMR techniques can identify different chemical groups of the SIS polymers. For easier comparison, HSQC ^{13}C-NMR and DEPT ^{13}C-NMR spectra of the control, initial SIS polymer before hydrogenation (HL = 0 %) and fully hydrogenated SIS polymer (HL > 99.9 %) are shown in **Figures S3-4**, respectively.

To accurately estimate the HL of SIS polymers, the peak locations of (1) unsaturated C = C bonds and (2) the resulting saturated C – C bonds need to be identified. Using the HSQC ^{13}C–NMR (see **Figure S4a**), unsaturated C = C bonds' peak (**k**) at $\delta =$ 5.1 ppm in the ^{1}H-NMR spectrum is also shown at $\delta =$ 124.8 ppm in the ^{13}C–NMR spectrum. The ^{1}H–NMR spectrum (see **Figure S2b**) clearly shows that the disappearance of C = C bonds' peak at complete hydrogenation (HL > 99.9 %). This information confirms the C = C bonds' peak location. In addition, with the DEPT ^{13}C-NMR (see **Figure S3**), the same C = C bonds' peak is positive, as expected for –CH groups attached to the unsaturated C = C bonds.

Similarly, the hydrogenated saturated C – C bonds' peak location (**h**) can be determined. Using the HSQC ^{13}C–NMR (see **Figure S4b**), the saturated C – C bonds' peak (**h**) at $\delta =$ 1.1 ppm in the ^{1}H-NMR spectrum is also found at $\delta =$ 37.5 ppm in the ^{13}C-NMR spectrum. The ^{1}H-NMR spectra (see **Figure S2**) show that the saturated C – C bonds' peak (**h**) increases with increasing HL. With the DEPT ^{13}C-NMR (see **Figure S3**), the same C – C bonds' peak is negative, as expected for $–CH_2$ groups originating from the saturated C – C bonds. In this manner, we can systematically confirm peak assignments for all chemical identities of the SIS polymers, analyze their chemical structures and evaluate the efficacy of our hydrogenation method.

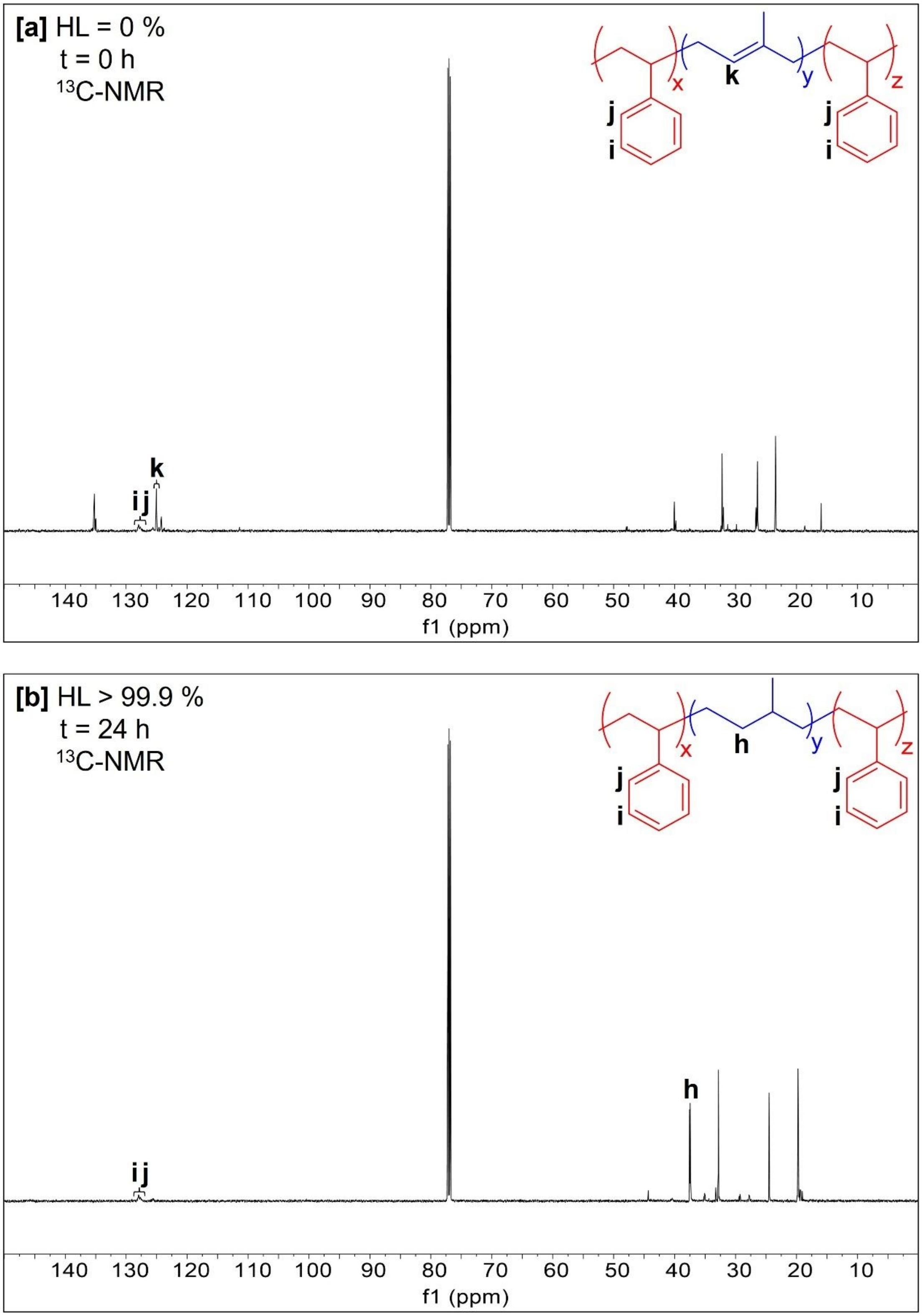

Figure S2. ^{13}C–NMR spectra of SIS polymers with **[a]** HL = 0 % (*t* = 1 h), and **[b]** HL > 99.9 % (*t* = 24 h). Chemical structures with peak assignments are shown.

S1.2.1 DEPT ^{13}C–NMR

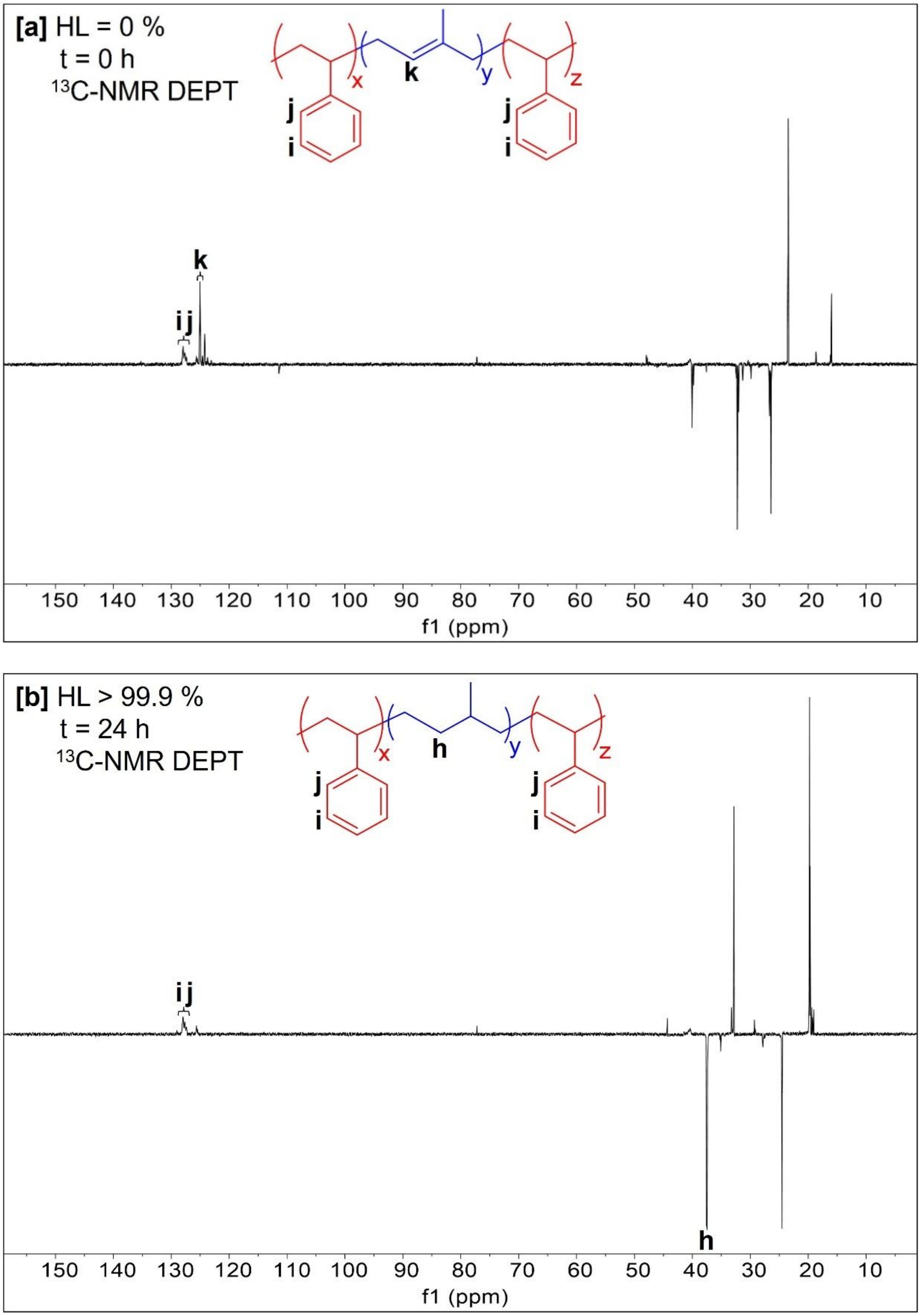

Figure S3. DEPT ^{13}C-NMR spectra of SIS polymers with **[a]** HL = 0 % (t = 1 h), and **[b]** HL > 99.9 % (t = 24 h). Chemical structures with peak assignments are shown.

S1.2.2 HSQC ^{13}C–NMR

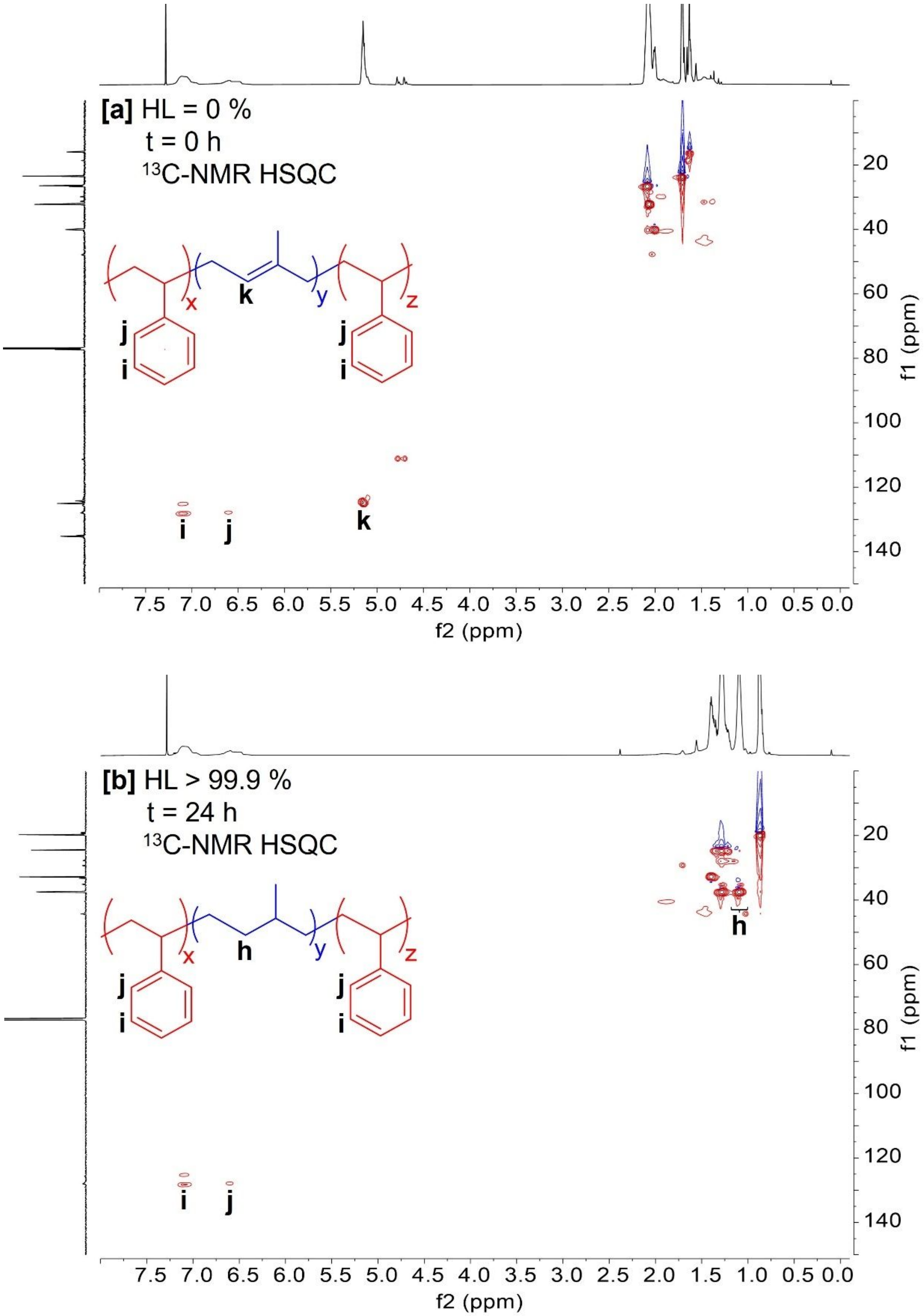

Figure S4. HSQC ^{13}C-NMR spectra of SIS polymers with **[a]** HL = 0 % (t = 1 h), and **[b]** HL > 99.9 % (t = 24 h). Chemical structures with peak assignments are shown.

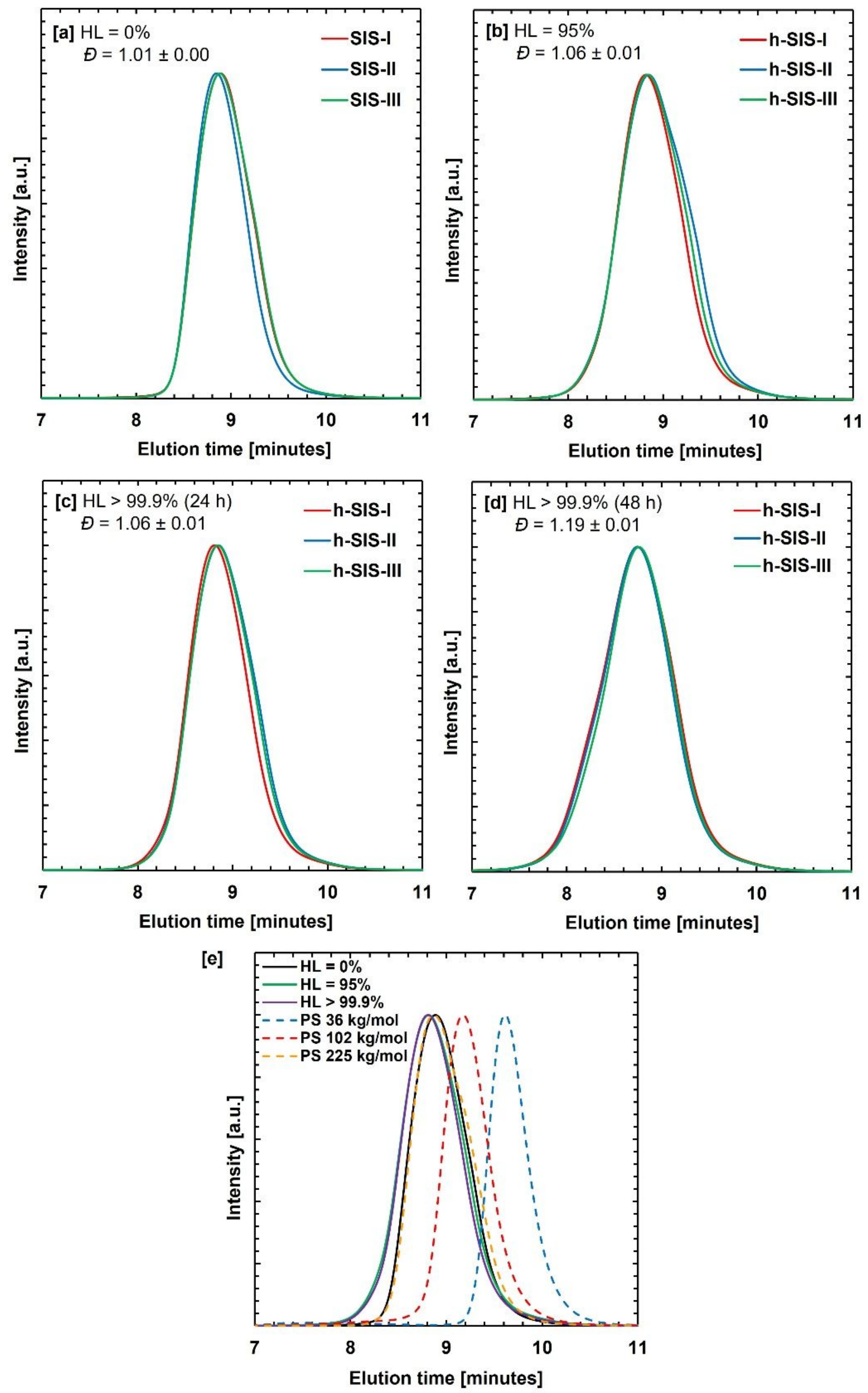

Figure S5. SEC trace of **[a]** control SIS (HL = 0 %) and hydrogenated polymer with **[b]** HL = 95 %, **[c]** HL > 99.9 % (24 h), and **[d]** HL > 99.9 % (48 h). At least 3 replicate samples were run to confirm data reproducibility in each set. **[e]** Combined SEC trace of control SIS and hydrogenated polymers with narrow-distribution (*Đ* = 1.00) PS standards of different molecular weights ($\overline{\mathrm{M}}_\mathrm{n}$ = 36, 102 and 225 kg/mol).

S2. Polymer film formation

S2.1 Method

To form a polymer film, a transparent, homogeneous polymer solution (5 wt% polymer) in toluene was prepared at room temperature. The polymer solution was then poured on a leveled Teflon plate (180 mL PTFE evaporating dishes, Thermo Fisher Scientific, Waltham, MA) in an oven. A 2–axis precision digital level checker (DWL-1300XY, Digi-Pas, Irvine, CA) was used to ensure the leveled film casting platform. The polymer film was formed via gradually evaporating the solvent at 80 °C for overnight. Next, the polymer film was soaked in deionized (DI) water (18.2 MΩ·cm, Millipore Direct-Q 5 UV system, Merck, Germany) at room temperature for 8 – 12 h to remove residual solvent while DI water was frequently replaced. Thickness of the polymer film was measured using a digital micrometer (H-2780, Mitutoyo, Japan), and a minimum of 3 – 6 measurements per each film were obtained.

S2.2 Results

Transparent, uniform–thickness thin films of SIS polymers with different hydrogenation levels were successfully prepared via solution cast method as shown in **Figure S6**. The initial SIS polymer film before hydrogenation (HL = 0 %, film thickness is 145 ± 6 μm) and fully hydrogenated SIS polymer film (HL > 99.9 %, film thickness is 118 ± 4 μm) are shown for brevity.

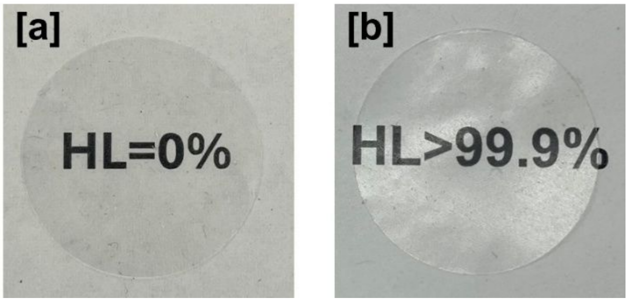

Figure S6. Successful formation of transparent, uniform–thickness SIS thin films with different hydrogenation levels (HL). **[a]** SIS film with HL = 0 %. **[b]** Hydrogenated SIS films with HL > 99.9 % are shown for brevity.

S3. References

(1) Bardet, M.; Foray, M. F.; Robert, D. Use of the dept pulse sequence to facilitate the 13c nmr structural analysis of lignins. *Die Makromolekulare Chemie: Macromolecular Chemistry and Physics* **1985**, *186* (7), 1495-1504. DOI: 10.1002/macp.1985.021860716.

(2) Doddrell, D.; Pegg, D.; Bendall, M. R. Distortionless enhancement of nmr signals by polarization transfer. *Journal of Magnetic Resonance (1969)* **1982**, *48* (2), 323-327. DOI: 10.1016/0022-2364(82)90286-4.

(3) Jiang, B.; Xiao, N.; Liu, H.; Zhou, Z.; Mao, X.-a.; Liu, M. Optimized quantitative dept and quantitative pommie experiments for 13c nmr. *Analytical Chemistry* **2008**, *80* (21), 8293-8298. DOI: 10.1021/ac8015455.

(4) Willker, W.; Flögel, U.; Leibfritz, D. Ultra-high-resolved hsqc spectra of multiple-13c-labeled biofluids. Academic Press: 1997; Vol. 125, pp 216-219.

(5) Reynolds, W. F.; McLean, S.; Tay, L. L.; Yu, M.; Enriquez, R. G.; Estwick, D. M.; Pascoe, K. O. Comparison of 13c resolution and sensitivity of hsqc and hmqc sequences and application of hsqc-based sequences to the total 1h and 13c spectral assignment of clionasterol. *Magnetic Resonance in Chemistry* **1997**, *35* (7), 455-462. DOI: 10.1002/(SICI)1097-458X(199707)35:7%3C455::AID-OMR116%3E3.0.CO;2-6.

(6) Bingol, K.; Li, D.-W.; Bruschweiler-Li, L.; Cabrera, O. A.; Megraw, T.; Zhang, F.; Brüschweiler, R. Unified and isomer-specific nmr metabolomics database for the accurate analysis of 13c–1h hsqc spectra. *ACS Chemical Biology* **2015**, *10* (2), 452-459. DOI: 10.1021/cb5006382.
